# Author Correction: Subjective socioeconomic status and income inequality are associated with self-reported morality across 67 countries

**DOI:** 10.1038/s41467-023-44402-9

**Published:** 2023-12-19

**Authors:** Christian T. Elbæk, Panagiotis Mitkidis, Lene Aarøe, Tobias Otterbring

**Affiliations:** 1 Department of Management, Aarhus University, 8210 Aarhus V, Denmark; 2 Social Science Research Institute, Duke University, 27701 Durham, NC USA; 3 Department of Political Science, Aarhus University, 8000 Aarhus C, Denmark; 4 Department of Management, University of Agder, 4630 Kristiansand, Norway

**Keywords:** Human behaviour, Society

Correction to: *Nature Communications* 10.1038/s41467-023-41007-0, published online 06 September 2023

The original version of this Article contained an error in two sentences which incorrectly stated ‘weekly’ instead of ‘daily’ on page 2 and page 10, respectively: “[…](4) Prosocial Intentions to Benefit Others, which captures the amount of monetary resources an individual reports being willing to donate to a national and international charity if given a weekly median income in one’s respective country” and “In this task, individuals were asked how much (in percent), if given a weekly median income, they would be willing to 1) keep to themselves, 2) donate to a national charity and 3) donate to an international charity”. The correct version states ‘daily’ in place of ‘weekly’ in both cases. This has been corrected in both the PDF and HTML versions of the Article.

The original version of the Supplementary Information associated with this Article contained an error in Supplementary Figures S3, S4, S5 and S6 in which the labels for the colouring were reversed in both the figures and the figure legends. In the correct version the colour red indicates *P* < 0.05 instead of *P* > 0.05, and the colour blue indicates *P* > 0.05 instead of *P* < 0.05.The HTML has been updated to include a corrected version of the Supplementary Information, which is also appended to this correction.

### Supplementary information

Supplementary Information

