# Supplementary material for: Subjective socioeconomic status and income inequality are associated with self-reported morality across 67 countries
Source: Nat Commun. 2023 Sep 6;14:5453. doi: 10.1038/s41467-023-41007-0 (PMC10482940; doi:10.1038/s41467-023-41007-0)
Supplement: Supplementary file 1 — Supplementary Information [file 41467_2023_41007_MOESM1_ESM.pdf]

Supplementary Information

Subjective Socioeconomic Status and Income Inequality is Associated with Self-Reported Morality Across 67 Countries

Table of Contents

1. Correlations......3

1.1. Region-level correlations [Multi-level]......3
1.1.1. Table S1......3

1.2. Correlations of dependent measures......5
1.2.1. Table S2......5
1.2.2. Table S3......5

2. Linear Mixed Effects Models......6

2.1. Imputed Data......6
2.1.1. Table S4......6

2.2 Representative Samples Only......7
2.2.1. Table S5......7
2.2.2. Figure S1......8
2.2.3. Figure S2......9

2.3. Within- & Between-Country SES Components......10
2.3.1. Table S6......10
2.3.2. Table S7......11
2.3.3. Table S8......12
2.3.4. Table S9......13

2.3 Models with Adjusted National Net-Income......14
2.3.1. Table S10......14

3. Nested Ordinary Least Squares Regression Results......15

3.1. Moral Identity......15
3.1.1. Table S11......15
3.1.2. Figure S3......17

3.2. Morality-as-Cooperation......18
3.2.1. Table S12......18
3.2.1. Figure S4......20

3.3 Moral Circle......21
3.3.1. Table S13......21
3.3.2. Figure S5......23

3.4. Prosocial Intention......24
3.3.1. Table S14......24
3.3.2. Figure S6......26

4. Cross Validations......27

4.1. All models......27
4.1.1. Table S15......27

5. Multilevel Confirmatory Factor Analysis Alignment......28

5.1. Moral Identity......28
5.1.1. Table S16......28
5.1.2. Table S17......29

5.1.3. Table S18 30
5.1.4. Table S19 31
5.1.5. Table S20 32
5.1.6. Table S21 33

5.2. Morality-as-Cooperation 34
5.2.1. Table S22 34
5.2.2. Table S23 35
5.2.3. Table S24 36
5.2.4. Table S25 37
5.2.5. Table S26 38
5.2.6. Table S27 39

6. Additional Descriptive Statistics 40

6.1. Summary statistics of subjective SES measure 40
6.1.1. Table S28 40
6.1.2. Table S29 41

1. Correlations

1.1. Region-level correlations [Multi-level]

1.1.1. Table S1

Table S1 | Region-level correlations

Region	X	Y	*r*	95% CI Low	95% CI High	*t*	*df*	*p*	*N*
Africa	SES	MI	0.012	-0.032	0.055	0.536	2040	1.000	2042
Africa	SES	MAC	-0.031	-0.074	0.011	-1.450	2126	1.000	2128
Africa	SES	MC	-0.040	-0.081	0.001	-1.935	2295	0.425	2297
Africa	SES	PI	-0.095	-0.137	-0.053	-4.428	2140	0.000	2142
Africa	SES	GINI	-0.194	-0.231	-0.158	-10.260	2681	0.000	2683
Africa	MI	MAC	0.376	0.338	0.413	18.175	2007	0.000	2009
Africa	MI	MC	0.007	-0.036	0.050	0.320	2084	1.000	2086
Africa	MI	PI	0.016	-0.029	0.061	0.681	1886	1.000	1888
Africa	MI	GINI	0.058	0.016	0.100	2.716	2165	0.060	2167
Africa	MAC	MC	0.019	-0.023	0.061	0.872	2148	1.000	2150
Africa	MAC	PI	-0.032	-0.077	0.012	-1.439	1964	1.000	1966
Africa	MAC	GINI	-0.002	-0.043	0.039	-0.084	2281	1.000	2283
Africa	MC	PI	0.088	0.045	0.130	3.991	2057	0.001	2059
Africa	MC	GINI	0.114	0.075	0.153	5.743	2486	0.000	2488
Africa	PI	GINI	0.082	0.042	0.123	3.965	2297	0.001	2299
Americas	SES	MI	-0.162	-0.183	-0.141	-14.779	8081	0.000	8083
Americas	SES	MAC	-0.092	-0.114	-0.070	-8.228	7964	0.000	7966
Americas	SES	MC	0.006	-0.016	0.027	0.522	8151	0.602	8153
Americas	SES	PI	-0.125	-0.147	-0.102	-10.800	7402	0.000	7404
Americas	SES	GINI	-0.114	-0.135	-0.092	-10.348	8177	0.000	8179
Americas	MI	MAC	0.272	0.252	0.293	25.275	7968	0.000	7970
Americas	MI	MC	0.050	0.028	0.072	4.527	8141	0.000	8143
Americas	MI	PI	0.062	0.039	0.084	5.320	7391	0.000	7393
Americas	MI	GINI	0.064	0.043	0.086	5.821	8197	0.000	8199
Americas	MAC	MC	0.079	0.058	0.101	7.142	8023	0.000	8025
Americas	MAC	PI	0.049	0.026	0.072	4.178	7274	0.000	7276
Americas	MAC	GINI	0.099	0.077	0.120	8.939	8081	0.000	8083
Americas	MC	PI	0.129	0.107	0.151	11.239	7472	0.000	7474
Americas	MC	GINI	0.075	0.053	0.096	6.809	8286	0.000	8288
Americas	PI	GINI	0.141	0.119	0.163	12.397	7571	0.000	7573
Asia	SES	MI	-0.166	-0.185	-0.146	-16.318	9409	0.000	9411
Asia	SES	MAC	-0.127	-0.147	-0.107	-12.460	9430	0.000	9432
Asia	SES	MC	-0.018	-0.038	0.002	-1.724	9547	0.170	9549
Asia	SES	PI	-0.097	-0.117	-0.077	-9.547	9578	0.000	9580
Asia	SES	GINI	-0.060	-0.080	-0.040	-5.892	9580	0.000	9582

Asia	MI	MAC	0.351	0.333	0.368	36.645	9576	0.000	9578
Asia	MI	MC	0.079	0.059	0.099	7.798	9640	0.000	9642
Asia	MI	PI	0.152	0.133	0.172	15.153	9677	0.000	9679
Asia	MI	GINI	0.080	0.060	0.100	7.936	9781	0.000	9783
Asia	MAC	MC	0.095	0.075	0.114	9.349	9661	0.000	9663
Asia	MAC	PI	0.140	0.120	0.159	13.922	9736	0.000	9738
Asia	MAC	GINI	0.193	0.174	0.212	19.484	9842	0.000	9844
Asia	MC	PI	0.161	0.142	0.181	16.215	9825	0.000	9827
Asia	MC	GINI	0.089	0.070	0.109	8.937	9953	0.000	9955
Asia	PI	GINI	-0.010	-0.029	0.010	-0.961	10081	0.337	10083
Europe	SES	MI	-0.083	-0.095	-0.070	-12.777	23793	0.000	23795
Europe	SES	MAC	0.010	-0.003	0.022	1.503	23908	0.133	23910
Europe	SES	MC	-0.015	-0.028	-0.003	-2.388	24137	0.034	24139
Europe	SES	PI	-0.077	-0.090	-0.065	-12.013	24009	0.000	24011
Europe	SES	GINI	0.054	0.042	0.067	8.486	24241	0.000	24243
Europe	MI	MAC	0.240	0.228	0.252	38.022	23684	0.000	23686
Europe	MI	MC	0.075	0.062	0.088	11.620	23803	0.000	23805
Europe	MI	PI	0.068	0.055	0.080	10.422	23696	0.000	23698
Europe	MI	GINI	0.034	0.021	0.047	5.276	23944	0.000	23946
Europe	MAC	MC	0.057	0.044	0.069	8.762	23912	0.000	23914
Europe	MAC	PI	0.083	0.071	0.096	12.911	23881	0.000	23883
Europe	MAC	GINI	0.145	0.133	0.157	22.752	24146	0.000	24148
Europe	MC	PI	0.096	0.084	0.109	15.010	24009	0.000	24011
Europe	MC	GINI	-0.019	-0.032	-0.007	-2.986	24254	0.008	24256
Europe	PI	GINI	0.078	0.066	0.091	12.262	24341	0.000	24343
Oceania	SES	MI	-0.286	-0.320	-0.250	-15.226	2610	0.000	2612
Oceania	SES	MAC	-0.171	-0.208	-0.134	-8.877	2616	0.000	2618
Oceania	SES	MC	0.027	-0.011	0.065	1.400	2627	0.808	2629
Oceania	SES	PI	-0.119	-0.156	-0.081	-6.123	2628	0.000	2630
Oceania	SES	GINI	-0.001	-0.039	0.037	-0.052	2628	1.000	2630
Oceania	MI	MAC	0.409	0.377	0.441	22.901	2605	0.000	2607
Oceania	MI	MC	0.030	-0.009	0.068	1.513	2611	0.783	2613
Oceania	MI	PI	0.153	0.116	0.190	7.925	2613	0.000	2615
Oceania	MI	GINI	0.023	-0.015	0.061	1.178	2613	0.955	2615
Oceania	MAC	MC	0.049	0.011	0.088	2.533	2617	0.079	2619
Oceania	MAC	PI	0.107	0.069	0.145	5.504	2618	0.000	2620
Oceania	MAC	GINI	-0.009	-0.047	0.030	-0.448	2618	1.000	2620
Oceania	MC	PI	0.076	0.038	0.114	3.918	2629	0.001	2631
Oceania	MC	GINI	-0.007	-0.045	0.032	-0.337	2629	1.000	2631
Oceania	PI	GINI	-0.105	-0.143	-0.067	-5.415	2631	0.000	2633

Notes: SES = Socioeconomic status, GINI = GINI coefficient, MI = Moral Identity, MAC = Morality-as-Cooperation, MC = Moral Circle, PI = Prosocial Intention. Multilevel Pearson correlations (two-sided) with region as a random effect. P-values adjusted for multiple comparison using Holm (1979).

1.2. Correlations of dependent measures

1.2.1. Table S2

Table S2 – Correlations between dependent variables

X	Y	*r*	95% CI Low	95% CI High	*t*	*df*	*p*	*N*
MI	MAC	0.286	0.278	0.295	63.965	45848	0	45850
MI	MC	0.070	0.061	0.079	15.122	46287	< .001	46289
MI	PI	0.078	0.069	0.087	16.719	45271	< .001	45273
MAC	MC	0.068	0.059	0.077	14.621	46369	< .001	46371
MAC	PI	0.086	0.077	0.095	18.474	45481	< .001	45483
MC	PI	0.118	0.109	0.127	25.418	46000	< .001	46002

Notes: MI = Moral Identity, MAC = Morality-as-Cooperation, MC = Moral Circle, PI = Prosocial Intention. Pearson correlations (two-sided). P-values adjusted for multiple comparison using Holm (1979).

1.2.2. Table S3

Table S3 | Correlations between Moral Circle and sub-measures of Prosocial Intention

X	Y	*r*	95% CI Low	95% CI High	*t*	*df*	*p*	*N*
MC	Nat.	-0.117	-0.126	-0.108	-25.220	46163	< 0.001	46165
MC	Int.	0.084	0.075	0.093	18.081	46190	< 0.001	46192
Nat.	Int.	-0.777	-0.780	-0.773	-267.511	47074	< 0.001	47076

Notes: MC = Moral Circle, Nat. = Prosocial Intention towards National charity, Int. = Prosocial Intention towards International charity. Pearson correlations (two-sided). P-values adjusted for multiple comparison using Holm (1979).

2. Linear Mixed Effects Models

2.1. Imputed Data

2.1.1. Table S4

Table S4 | Multilevel models using imputed data

	**Moral Identity**			**Morality-as-Cooperation**			**Moral Circle**			**Prosocial Behavior**		
Predictors	*β* (*95% CI*)	*t* (*df*)	*p*	*β* (*95% CI*)	*t* (*df*)	*p*	*β* (*95% CI*)	*t* (*df*)	*p*	*β* (*95% CI*)	*t* (*df*)	*p*
Subjective SES	-0.14 (-0.15 – -0.13)	-5.81 (47633)	**<0.001**	-0.07 (-0.08 – -0.06)	-0.69 (47633)	**<0.001**	0.01 (0.00 – 0.02)	1.11 (47633)	**0.005**	-0.08 (-0.09 – -0.08)	-2.89 (47633)	**<0.001**
GINI Index	0.13 (0.06 – 0.21)	3.29 (47633)	**0.001**	0.03 (-0.04 – 0.11)	1.66 (47633)	0.360	0.08 (0.03 – 0.13)	2.97 (47633)	**0.003**	0.05 (-0.03 – 0.12)	1.47 (47633)	0.207
Gender [Female]	0.12 (0.10 – 0.13)	13.27 (47633)	**<0.001**	0.09 (0.07 – 0.11)	10.04 (47633)	**<0.001**	0.17 (0.15 – 0.19)	18.49 (47633)	**<0.001**	0.15 (0.13 – 0.17)	17.09 (47633)	**<0.001**
Age	0.04 (0.03 – 0.05)	8.59 (47633)	**<0.001**	0.02 (0.01 – 0.02)	3.18 (47633)	**0.001**	0.07 (0.06 – 0.08)	14.25 (47633)	**<0.001**	0.05 (0.04 – 0.05)	9.93 (47633)	**<0.001**
SES x GINI	-0.00 (-0.01 – 0.01)	-0.26 (47633)	0.794	-0.01 (-0.02 – -0.00)	-2.28 (47633)	**0.023**	-0.00 (-0.01 – 0.01)	-0.57 (47633)	0.572	-0.00 (-0.01 – 0.01)	-0.81 (47633)	0.416
**Random Effects**												
σ^2	173.06			119.52			25.72			1071.52		
τ_{00}	20.56 $_{country}$			11.33 $_{country}$			1.18 $_{country}$			118.86 $_{country}$		
ICC	0.11			0.09			0.04			0.10		
N	67 $_{country}$			67 $_{country}$			67 $_{country}$			67 $_{country}$		
Observations	47641			47641			47641			47641		
Marginal R^2/ Conditional R^2	0.043 / 0.144			0.008 / 0.094			0.017 / 0.060			0.017 / 0.115		
AIC	381026.416			363379.282			290150.897			467870.468		

Notes: All models are linear mixed-effects models (two-sided).

2.2 Representative Samples Only

2.2.1. Table S5

Table S5 | Multilevel models using only nationally representative samples

	Moral Identity			Morality-as-Cooperation			Moral Circle			Prosocial Intention		
Predictors	*β (95% CI)*	*t (df)*	*p*	*β (95% CI)*	*t (df)*	*p*	*β (95% CI)*	*t (df)*	*p*	*β (95% CI)*	*t (df)*	*p*
Subjective SES	-0.17 (-0.18 – -0.16)	4.52 (27948)	**<0.001**	-0.08 (-0.09 – -0.07)	4.31 (28052)	**<0.001**	0.02 (0.01 – 0.03)	-0.31 (28289)	**<0.001**	-0.09 (-0.11 – -0.08)	-2.77 (28317)	**<0.001**
GINI Index	0.22 (0.12 – 0.31)	6.83 (27948)	**<0.001**	0.13 (0.03 – 0.22)	4.70 (28052)	**0.007**	0.03 (-0.04 – 0.10)	0.40 (28289)	0.387	0.01 (-0.10 – 0.12)	0.02 (28317)	0.873
Gender[Female]	0.13 (0.11 – 0.16)	11.86 (27948)	**<0.001**	0.09 (0.07 – 0.11)	7.57 (28052)	**<0.001**	0.17 (0.15 – 0.20)	14.82 (28289)	**<0.001**	0.14 (0.12 – 0.16)	12.43 (28317)	**<0.001**
Age	0.02 (0.01 – 0.03)	3.01 (27948)	**0.003**	0.02 (0.01 – 0.03)	3.10 (28052)	**0.002**	0.05 (0.04 – 0.06)	8.81 (28289)	**<0.001**	0.06 (0.05 – 0.07)	10.31 (28317)	**<0.001**
SES x GINI	-0.05 (-0.06 – -0.04)	-8.82 (27948)	**<0.001**	-0.04 (-0.05 – -0.03)	-6.25 (28052)	**<0.001**	0.01 (-0.01 – 0.02)	0.84 (28289)	0.400	0.00 (-0.01 – 0.01)	0.50 (28317)	0.617
**Random Effects**												
σ^2	171.47			117.37			25.62			1112.33		
τ_{00}	15.04 $_{country\_name}$			8.22 $_{country\_name}$			0.98 $_{country\_name}$			131.37 $_{country\_name}$		
ICC	0.08			0.07			0.04			0.11		
N	28 $_{country\_name}$			28 $_{country\_name}$			28 $_{country\_name}$			28 $_{country\_name}$		
Observations	27956			28060			28297			28325		
Marginal R^2 / Conditional R^2	0.076 / 0.150			0.023 / 0.087			0.011 / 0.047			0.016 / 0.120		
AIC	223308.308			213497.600			172227.290			279213.302		

Notes: All models are linear mixed-effects models (two-sided).

2.2.2. Figure S1

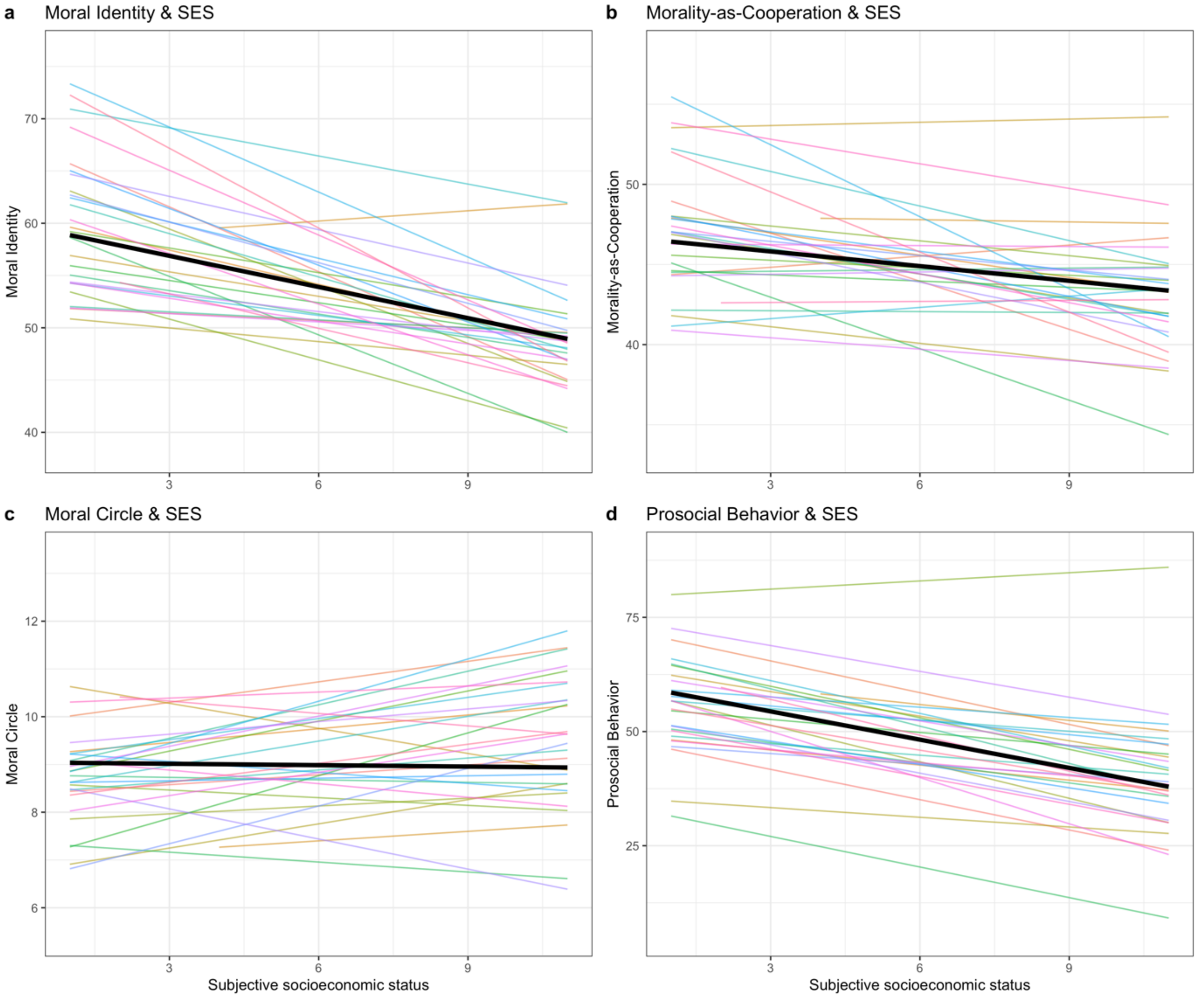

Fig. S1 | Within-country and between-country associations between Moral Identity, Morality-as-Cooperation, size of Moral Circle, Prosocial Intentions, and Subjective Socioeconomic Status (SES) for countries with nationally representative samples. For all four panels (a, b, c, d), colored lines indicate within-country associations highlighting a main pattern where most associations are negative, while simultaneously outlining the degree of heterogeneity between-countries, as a selection of within-country associations are positive. The bolded black line for each panel indicates the overall relationship across the 67 countries. **a**, Association between SES and individual-level Moral Identity. **b**, Association between SES and Morality-as-Cooperation. **c**, Association between SES and the size of one's Moral Circle, where size indicates the circle of people or other entities for which one is concerned whether right or wrong is done toward them. **d**, Association between SES and Prosocial Intentions, measured as the amount of money (out of a median income) one would be willing to donate to a national and international charity.

2.2.3. Figure S2

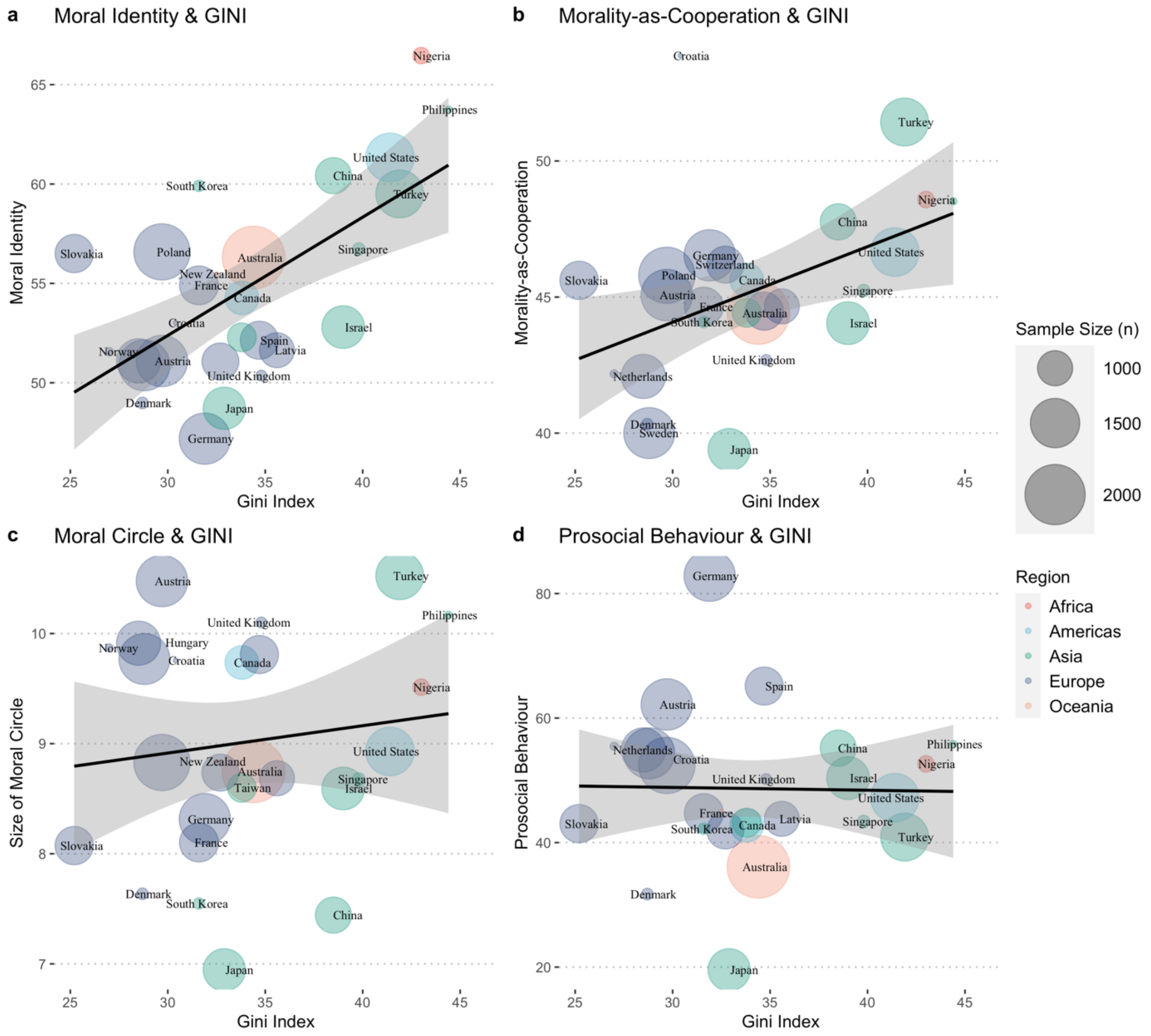

Fig. S2 | Country and region-level relationships between Moral Identity, Morality-as-Cooperation, size of Moral Circle, Prosocial Intentions, and level of Income Inequality (GINI) for countries with nationally representative samples. a, Association between GINI and Moral Identity. **b**, Association between GINI and Morality-as-Cooperation. **c**, Association between GINI and the size of one's Moral Circle. **d**, Association between GINI and Prosocial Intentions (willingness to donate to a national and international charity). The grey shade area, in all panels, represents the 95% confidence interval.

2.3. *Within- & Between-Country SES Components*

2.3.1. *Table S6*

Table S6 | Multilevel model for moral identity split into within- & between-country components

	Moral Identity								
Predictors	*β (95% CI)*	*t (df)*	*p*	*β (95% CI)*	*t (df)*	*p*	*β (95% CI)*	*t (df)*	*p*
SES (within- & between-country effect)	-0.14 (-0.15 – -0.13)	-29.95 (45833)	**<0.001**						
GINI	0.13 (0.04 – 0.21)	3.02 (45833)	**0.002**	0.14 (0.06 – 0.23)	3.44 (45833)	**0.001**	0.15 (0.06 – 0.24)	3.37 (45895)	**0.001**
Gender[Female]	0.12 (0.10 – 0.14)	13.33 (45833)	**<0.001**	0.12 (0.10 – 0.14)	13.33 (45833)	**<0.001**	0.11 (0.09 – 0.13)	12.00 (45895)	**<0.001**
Age	0.04 (0.03 – 0.05)	8.45 (45833)	**<0.001**	0.04 (0.03 – 0.05)	8.45 (45833)	**<0.001**	0.04 (0.03 – 0.05)	8.68 (45895)	**<0.001**
SES (within-country effect)				-0.13 (-0.14 – -0.12)	-29.98 (45833)	**<0.001**			
SES (between-subject effect)							0.02 (-0.06 – 0.11)	0.51 (45895)	0.610
**Random Effects**									
σ^2	177.00			177.00			180.51		
τ_{00}	24.94 country_name			24.21 country_name			24.48 country_name		
ICC	0.12			0.12			0.12		
N	67 country_name			67 country_name			67 country_name		
Observations	45840			45840			45902		
Marginal R^2 / Conditional R^2	0.040 / 0.158			0.040 / 0.156			0.025 / 0.141		
AIC	367664.230			367662.458			369051.673		

Notes: All models are linear mixed-effects models (two-sided).

2.3.2. Table S7

Table S7 | Multilevel model for Morality-as-Cooperation split into within- & between-country components

	Morality-as-Cooperation								
Predictors	*β (95% CI)*	*t (df)*	*p*	*β (95% CI)*	*t (df)*	*p*	*β (95% CI)*	*t (df)*	*p*
SES (within- & between-country effect)	-0.07 (-0.08 – -0.06)	-14.59 (45947)	**<0.001**						
GINI	0.03 (-0.04 – 0.10)	0.77 (45947)	0.440	0.04 (-0.04 – 0.11)	0.99 (45947)	0.323	0.05 (-0.03 – 0.13)	1.30 (46022)	0.193
Gender [Female]	0.09 (0.07 – 0.11)	10.06 (45947)	**<0.001**	0.09 (0.07 – 0.11)	10.06 (45947)	**<0.001**	0.09 (0.07 – 0.10)	9.48 (46022)	**<0.001**
Age	0.02 (0.01 – 0.02)	3.19 (45947)	**0.001**	0.02 (0.01 – 0.02)	3.20 (45947)	**0.001**	0.02 (0.01 – 0.03)	3.35 (46022)	**0.001**
SES (within-country effect)				-0.07 (-0.07 – -0.06)	-14.63 (45947)	**<0.001**			
SES (between-subject effect)							0.04 (-0.04 – 0.12)	1.02 (46022)	0.309
**Random Effects**									
σ^2	122.31			122.31			122.97		
τ_{00}	13.03 $_{\text{country\_name}}$			12.77 $_{\text{country\_name}}$			12.74 $_{\text{country\_name}}$		
ICC	0.10			0.09			0.09		
N	67 $_{\text{country\_name}}$			67 $_{\text{country\_name}}$			67 $_{\text{country\_name}}$		
Observations	45954			45954			46029		
Marginal R^2 / Conditional R^2	0.008 / 0.103			0.008 / 0.101			0.005 / 0.099		
AIC	351576.106			351574.818			352389.532		

Notes: All models are linear mixed-effects models (two-sided).

2.3.3. Table S8

Table S8 | Multilevel model for Moral Circle split into within- & between-country components

	Moral Circle								
Predictors	*β (95% CI)*	*t (df)*	*p*	*β (95% CI)*	*t (df)*	*p*	*β (95% CI)*	*t (df)*	*p*
SES (within- & between-country effect)	0.01 (0.00 – 0.02)	2.92 (46647)	**0.003**						
GINI	0.08 (0.03 – 0.13)	3.01 (46647)	**0.003**	0.08 (0.03 – 0.13)	2.97 (46647)	**0.003**	0.05 (-0.00 – 0.11)	1.94 (46712)	0.052
Gender [Female]	0.17 (0.15 – 0.19)	18.45 (46647)	**<0.001**	0.17 (0.15 – 0.19)	18.45 (46647)	**<0.001**	0.17 (0.15 – 0.19)	18.53 (46712)	**<0.001**
Age	0.07 (0.06 – 0.08)	14.01 (46647)	**<0.001**	0.07 (0.06 – 0.08)	14.01 (46647)	**<0.001**	0.07 (0.06 – 0.08)	13.93 (46712)	**<0.001**
SES (within-country effect)				0.01 (0.00 – 0.02)	3.06 (46647)	**0.002**			
SES (between-subject effect)							-0.07 (-0.13 – -0.02)	-2.89 (46712)	**0.004**
**Random Effects**									
σ^2	26.08			26.08			26.09		
τ_{00}	1.23 $_{\text{country\_name}}$			1.21 $_{\text{country\_name}}$			1.06 $_{\text{country\_name}}$		
ICC	0.04			0.04			0.04		
N	67 $_{\text{country\_name}}$			67 $_{\text{country\_name}}$			67 $_{\text{country\_name}}$		
Observations	46654			46654			46719		
Marginal R^2 / Conditional R^2	0.017 / 0.061			0.017 / 0.060			0.021 / 0.059		
AIC	284795.027			284794.182			285198.733		

Notes: All models are linear mixed-effects models (two-sided).

2.3.4. *Table S9*

Table S9 | Multilevel model for Prosocial Intention split into within- & between-country components

	Prosocial Intention								
Predictors	*β (95% CI)*	*t (df)*	*p*	*β (95% CI)*	*t (df)*	*p*	*β (95% CI)*	*t (df)*	*p*
SES (within- & between-country effect)	-0.08 (-0.09 – -0.07)	-18.06 (45650)	**<0.001**						
GINI	0.06 (-0.01 – 0.14)	1.59 (45650)	0.112	0.07 (-0.01 – 0.15)	1.81 (45650)	0.071	0.05 (-0.03 – 0.13)	1.25 (45743)	0.211
Gender [Female]	0.15 (0.13 – 0.17)	16.88 (45650)	**<0.001**	0.15 (0.13 – 0.17)	16.88 (45650)	**<0.001**	0.14 (0.13 – 0.16)	16.18 (45743)	**<0.001**
Age	0.05 (0.04 – 0.06)	10.04 (45650)	**<0.001**	0.05 (0.04 – 0.06)	10.05 (45650)	**<0.001**	0.05 (0.04 – 0.06)	10.18 (45743)	**<0.001**
SES (within-country effect)				-0.08 (-0.09 – -0.07)	-18.03 (45650)	**<0.001**			
SES (between-subject effect)							-0.05 (-0.13 – 0.03)	-1.31 (45743)	0.190
**Random Effects**									
σ^2	1104.55			1104.56			1112.29		
τ_{00}	131.69 $_{country\_name}$			134.16 $_{country\_name}$			132.44 $_{country\_name}$		
ICC	0.11			0.11			0.11		
N	67 $_{country\_name}$			67 $_{country\_name}$			67 $_{country\_name}$		
Observations	45657			45657			45750		
Marginal R^2 / Conditional R^2	0.018 / 0.123			0.018 / 0.124			0.014 / 0.119		
AIC	449772.325			449773.507			451000.447		

Notes: All models are linear mixed-effects models (two-sided).

2.3 Models with Adjusted National Net-Income
2.3.1. Table S10

Table S10 | Multilevel models including Adjusted Net Income

	Moral Identity			Morality-as-Cooperation			Moral Circle			Prosocial Behavior		
Predictors	*β* *(95% CI)*	*t* *(df)*	*p*	*β* *(95% CI)*	*t* *(df)*	*p*	*β* *(95% CI)*	*t* *(df)*	*p*	*β* *(95% CI)*	*t* *(df)*	*p*
Subjective SES	-0.14 (-0.15 – -0.13)	-5.49 (44011)	**<0.001**	-0.07 (-0.08 – -0.06)	-0.39 (44121)	**<0.001**	0.02 (0.01 – 0.02)	1.18 (44814)	**0.002**	-0.09 (-0.09 – -0.08)	-2.72 (43817)	**<0.001**
GINI Index	0.08 (-0.01 – 0.18)	1.77 (44011)	0.083	-0.02 (-0.10 – 0.06)	0.32 (44121)	0.619	0.06 (-0.00 – 0.12)	1.94 44814)	0.064	-0.00 (-0.09 – 0.08)	0.19 (43817)	0.940
Adjusted net income	-0.10 (-0.20 – -0.00)	-1.98 (44011)	**0.048**	-0.11 (-0.20 – -0.02)	-2.43 (44121)	**0.015**	-0.05 (-0.11 – 0.01)	-1.65 (44814)	0.100	-0.14 (-0.23 – -0.05)	-3.13 (43817)	**0.002**
Gender [Female]	0.13 (0.11 – 0.14)	13.81 (44011)	**<0.001**	0.09 (0.08 – 0.11)	10.17 (44121)	**<0.001**	0.17 (0.15 – 0.19)	18.35 (44814)	**<0.001**	0.15 (0.13 – 0.17)	16.86 (43817)	**<0.001**
Age	0.04 (0.03 – 0.05)	8.23 (44011)	**<0.001**	0.01 (0.00 – 0.02)	2.72 (44121)	**0.006**	0.07 (0.06 – 0.08)	13.81 (44814)	**<0.001**	0.05 (0.04 – 0.06)	9.78 (43817)	**<0.001**
SES x GINI	-0.00 (-0.01 – 0.01)	-0.35 (44011)	0.730	-0.01 (-0.02 – -0.00)	-2.55 (44121)	**0.011**	-0.00 (-0.01 – 0.01)	-0.56 (44814)	0.575	-0.00 (-0.01 – 0.01)	-0.84 (43817)	0.400
**Random Effects**												
σ^2	176.29			122.21			26.00			1106.52		
τ_{00}	24.20 country_name			12.05 country_name			1.18 country_name			117.87 country_name		
ICC	0.12			0.09			0.04			0.10		
N	65 country_name			65 country_name			65 country_name			65 country_name		
Observations	44020			44130			44823			43826		
Marginal R^2 / Conditional R^2	0.045 / 0.161			0.016 / 0.104			0.018 / 0.061			0.031 / 0.124		
AIC	352918.923			337614.006			273517.482			431833.528		

Please Note: All models are linear mixed-effects models (two-sided). GINI and Adjusted Net Income are highly correlated (t(48561) = -105.54, r = -.43, p < .001), resulting in the Adjusted Net Income measure capturing a substantial part of the variation attributed to the GINI index in previous models.

3. Nested Ordinary Least Squares Regression Results

3.1. Moral Identity

3.1.1. Table S11

Table S11 | Nested OLS regression results

Country	***DV***	***IV***	***Estimate***	***SE***	***t***	***df***	***p***
Argentina	MI	SES	-0.385	0.326	-1.181	719	0.238
Australia	MI	SES	-2.066	0.143	-14.409	2122	0.000
Austria	MI	SES	-0.229	0.210	-1.090	1586	0.276
Bangladesh	MI	SES	-1.191	0.366	-3.251	586	0.001
Belgium	MI	SES	-0.404	0.213	-1.895	1150	0.058
Bolivia	MI	SES	-3.538	0.983	-3.600	27	0.001
Brazil	MI	SES	-0.719	0.179	-4.024	2211	0.000
Bulgaria	MI	SES	-1.191	0.400	-2.976	655	0.003
Canada	MI	SES	-1.095	0.219	-4.996	954	0.000
Chile	MI	SES	-0.814	0.787	-1.035	95	0.304
China	MI	SES	0.329	0.252	1.306	1028	0.192
Colombia	MI	SES	-1.026	0.267	-3.838	1261	0.000
Costa Rica	MI	SES	-1.665	1.855	-0.897	23	0.379
Croatia	MI	SES	-0.782	0.336	-2.328	509	0.020
Cuba	MI	SES	0.409	1.070	0.382	41	0.705
Denmark	MI	SES	-0.435	0.343	-1.271	551	0.204
Dominican Republic	MI	SES	-0.891	1.202	-0.741	34	0.464
Ecuador	MI	SES	-0.053	0.722	-0.073	146	0.942
El Salvador	MI	SES	-5.564	2.137	-2.604	26	0.015
Finland	MI	SES	-0.774	0.233	-3.321	660	0.001
France	MI	SES	-1.823	0.213	-8.566	1113	0.000
Germany	MI	SES	-1.302	0.223	-5.829	1579	0.000
Ghana	MI	SES	-0.368	0.905	-0.407	388	0.684
Greece	MI	SES	-0.638	0.290	-2.201	634	0.028
Guatemala	MI	SES	-3.009	1.292	-2.328	46	0.024
Honduras	MI	SES	1.354	1.310	1.034	22	0.313
Hungary	MI	SES	-0.787	0.386	-2.040	504	0.042
India	MI	SES	-0.775	0.280	-2.764	722	0.006
Iraq	MI	SES	0.248	0.230	1.078	1126	0.282
Ireland	MI	SES	0.073	0.266	0.275	772	0.783
Israel	MI	SES	-0.711	0.194	-3.667	1243	0.000
Italy	MI	SES	-0.933	0.209	-4.474	1270	0.000
Japan	MI	SES	-1.864	0.181	-10.292	1228	0.000

Latvia	MI	SES	-0.746	0.219	-3.408	993	0.001
Mexico	MI	SES	-0.498	0.242	-2.055	1301	0.040
Morocco	MI	SES	-0.362	0.348	-1.040	793	0.299
Nepal	MI	SES	-0.997	0.374	-2.668	555	0.008
Netherlands	MI	SES	-0.255	0.198	-1.283	1295	0.200
New Zealand	MI	SES	-1.383	0.276	-5.011	507	0.000
Nicaragua	MI	SES	-3.090	1.765	-1.751	14	0.102
Nigeria	MI	SES	-0.897	0.290	-3.093	604	0.002
North Macedonia	MI	SES	-0.943	0.308	-3.065	713	0.002
Norway	MI	SES	-0.620	0.289	-2.144	524	0.033
Pakistan	MI	SES	-0.465	0.330	-1.408	554	0.160
Panama	MI	SES	-1.166	2.192	-0.532	16	0.602
Paraguay	MI	SES	2.735	2.202	1.242	14	0.235
Peru	MI	SES	0.986	0.841	1.172	89	0.244
Philippines	MI	SES	-2.072	0.334	-6.199	520	0.000
Poland	MI	SES	-1.160	0.173	-6.709	1803	0.000
Romania	MI	SES	-0.375	0.256	-1.464	998	0.143
Russia	MI	SES	-1.488	0.377	-3.951	505	0.000
Senegal	MI	SES	0.211	0.420	0.503	543	0.615
Serbia	MI	SES	-0.461	0.307	-1.502	1063	0.133
Singapore	MI	SES	-1.811	0.294	-6.166	558	0.000
Slovakia	MI	SES	-1.297	0.276	-4.701	1094	0.000
South Africa	MI	SES	0.828	0.309	2.681	925	0.008
South Korea	MI	SES	-1.063	0.246	-4.324	548	0.000
Spain	MI	SES	-0.567	0.232	-2.447	1088	0.015
Sweden	MI	SES	-0.268	0.179	-1.495	1566	0.135
Switzerland	MI	SES	-0.729	0.218	-3.339	1051	0.001
Taiwan	MI	SES	-1.617	0.274	-5.893	831	0.000
Turkey	MI	SES	-2.067	0.192	-10.738	1437	0.000
Ukraine	MI	SES	-2.044	0.378	-5.411	575	0.000
United Kingdom	MI	SES	-1.098	0.297	-3.694	544	0.000
United States	MI	SES	-2.546	0.169	-15.066	1469	0.000
Uruguay	MI	SES	0.302	1.572	0.192	47	0.848
Venezuela	MI	SES	-1.184	0.768	-1.542	94	0.126

Note: MI = Moral Identity, SES = Subjective Socioeconomic Status. Model is a Nested Ordinary Least Square (OLS) regression (two-sided). Each row indicates a separate OLS regression.

3.1.2. Figure S3

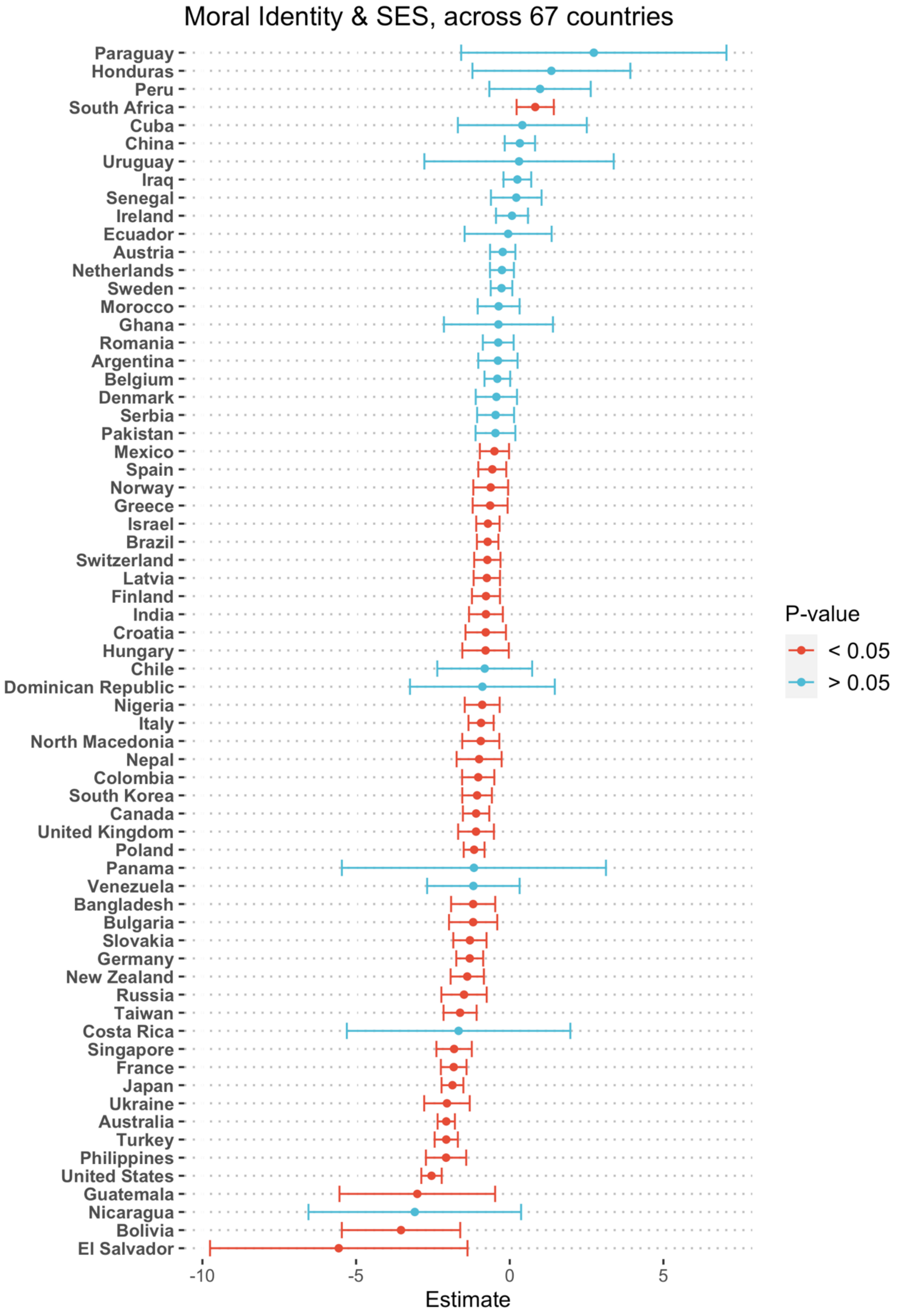

Fig. S3 | Nested OLS regression estimates for associations between Moral Identity and Subjective Socioeconomic Status (SES) across all 67 countries. Dots indicate OLS regression estimates and error bars indicate 95% confidence intervals. Dots and errors bars in red indicate regression estimates with p-values < 0.05. Dots and error bars in light blue indicate regression estimates with p-values > 0.05. Ordinary Least Squares regressions (two-sided) for each of the 67 countries (i.e., Nested OLS). *N* = 50,563 survey participants.

3.2. Morality-as-Cooperation

3.2.1. Table S12

Table S12 | Nested OLS regression results

Country	*DV*	*IV*	*Estimate*	*SE*	*t*	*df*	*p*
Argentina	MAC	SES	0.005	0.255	0.019	719	0.985
Australia	MAC	SES	-1.000	0.112	-8.896	2122	0.000
Austria	MAC	SES	0.231	0.192	1.204	1586	0.229
Bangladesh	MAC	SES	-0.606	0.267	-2.272	586	0.024
Belgium	MAC	SES	-0.180	0.181	-0.997	1150	0.319
Bolivia	MAC	SES	-0.605	0.971	-0.623	27	0.538
Brazil	MAC	SES	-0.420	0.160	-2.626	2211	0.009
Bulgaria	MAC	SES	0.004	0.302	0.014	655	0.989
Canada	MAC	SES	-0.464	0.192	-2.414	954	0.016
Chile	MAC	SES	0.143	0.589	0.242	95	0.809
China	MAC	SES	-0.045	0.229	-0.197	1028	0.844
Colombia	MAC	SES	-0.265	0.257	-1.031	1261	0.303
Costa Rica	MAC	SES	-0.118	1.641	-0.072	23	0.943
Croatia	MAC	SES	0.067	0.268	0.251	509	0.802
Cuba	MAC	SES	-0.973	0.614	-1.586	41	0.120
Denmark	MAC	SES	-0.346	0.293	-1.181	551	0.238
Dominican Republic	MAC	SES	-0.883	1.339	-0.659	34	0.514
Ecuador	MAC	SES	-0.137	0.502	-0.272	146	0.786
El Salvador	MAC	SES	0.154	1.484	0.104	26	0.918
Finland	MAC	SES	0.194	0.177	1.100	660	0.272
France	MAC	SES	-0.495	0.155	-3.189	1113	0.001
Germany	MAC	SES	-0.309	0.172	-1.792	1579	0.073
Ghana	MAC	SES	-0.442	0.670	-0.659	388	0.510
Greece	MAC	SES	-0.504	0.227	-2.219	634	0.027
Guatemala	MAC	SES	-1.761	1.060	-1.661	46	0.104
Honduras	MAC	SES	-0.848	1.151	-0.736	22	0.469
Hungary	MAC	SES	-0.156	0.254	-0.616	504	0.538
India	MAC	SES	-1.656	0.258	-6.409	722	0.000
Iraq	MAC	SES	-0.156	0.211	-0.736	1126	0.462
Ireland	MAC	SES	0.152	0.262	0.579	772	0.563
Israel	MAC	SES	-0.130	0.200	-0.648	1243	0.517
Italy	MAC	SES	-0.419	0.148	-2.832	1270	0.005
Japan	MAC	SES	-1.071	0.117	-9.114	1228	0.000
Latvia	MAC	SES	0.033	0.188	0.176	993	0.860
Mexico	MAC	SES	-0.211	0.195	-1.081	1301	0.280
Morocco	MAC	SES	-0.236	0.241	-0.979	793	0.328
Nepal	MAC	SES	-1.128	0.355	-3.178	555	0.002

Netherlands	MAC	SES	-0.018	0.166	-0.109	1295	0.913
New Zealand	MAC	SES	-0.527	0.256	-2.061	507	0.040
Nicaragua	MAC	SES	-1.672	0.833	-2.008	14	0.064
Nigeria	MAC	SES	-0.719	0.289	-2.484	604	0.013
North Macedonia	MAC	SES	-0.933	0.305	-3.062	713	0.002
Norway	MAC	SES	0.234	0.259	0.902	524	0.368
Pakistan	MAC	SES	-0.092	0.261	-0.352	554	0.725
Panama	MAC	SES	-0.892	1.408	-0.634	16	0.535
Paraguay	MAC	SES	-1.738	0.996	-1.745	14	0.103
Peru	MAC	SES	-0.524	0.589	-0.891	89	0.375
Philippines	MAC	SES	-1.496	0.301	-4.967	520	0.000
Poland	MAC	SES	-0.404	0.143	-2.826	1803	0.005
Romania	MAC	SES	0.171	0.181	0.946	998	0.344
Russia	MAC	SES	-0.456	0.309	-1.476	505	0.141
Senegal	MAC	SES	0.101	0.328	0.307	543	0.759
Serbia	MAC	SES	-0.559	0.279	-2.002	1063	0.046
Singapore	MAC	SES	-0.624	0.242	-2.577	558	0.010
Slovakia	MAC	SES	-0.296	0.203	-1.458	1094	0.145
South Africa	MAC	SES	0.124	0.334	0.370	925	0.712
South Korea	MAC	SES	-0.623	0.195	-3.196	548	0.001
Spain	MAC	SES	0.048	0.201	0.237	1088	0.813
Sweden	MAC	SES	-0.237	0.154	-1.542	1566	0.123
Switzerland	MAC	SES	-0.019	0.174	-0.112	1051	0.911
Taiwan	MAC	SES	-0.598	0.247	-2.417	831	0.016
Turkey	MAC	SES	-0.512	0.143	-3.569	1437	0.000
Ukraine	MAC	SES	-0.285	0.302	-0.944	575	0.345
United Kingdom	MAC	SES	0.022	0.255	0.087	544	0.931
United States	MAC	SES	-1.251	0.135	-9.281	1469	0.000
Uruguay	MAC	SES	1.089	1.285	0.847	47	0.401
Venezuela	MAC	SES	-0.226	0.498	-0.454	94	0.651

Notes: MAC = Morality-as-Cooperation, SES = Subjective Socioeconomic Status. Model is a Nested Ordinary Least Square (OLS) regression (two-sided). Each row indicates a separate OLS regression.

3.2.1. Figure S4

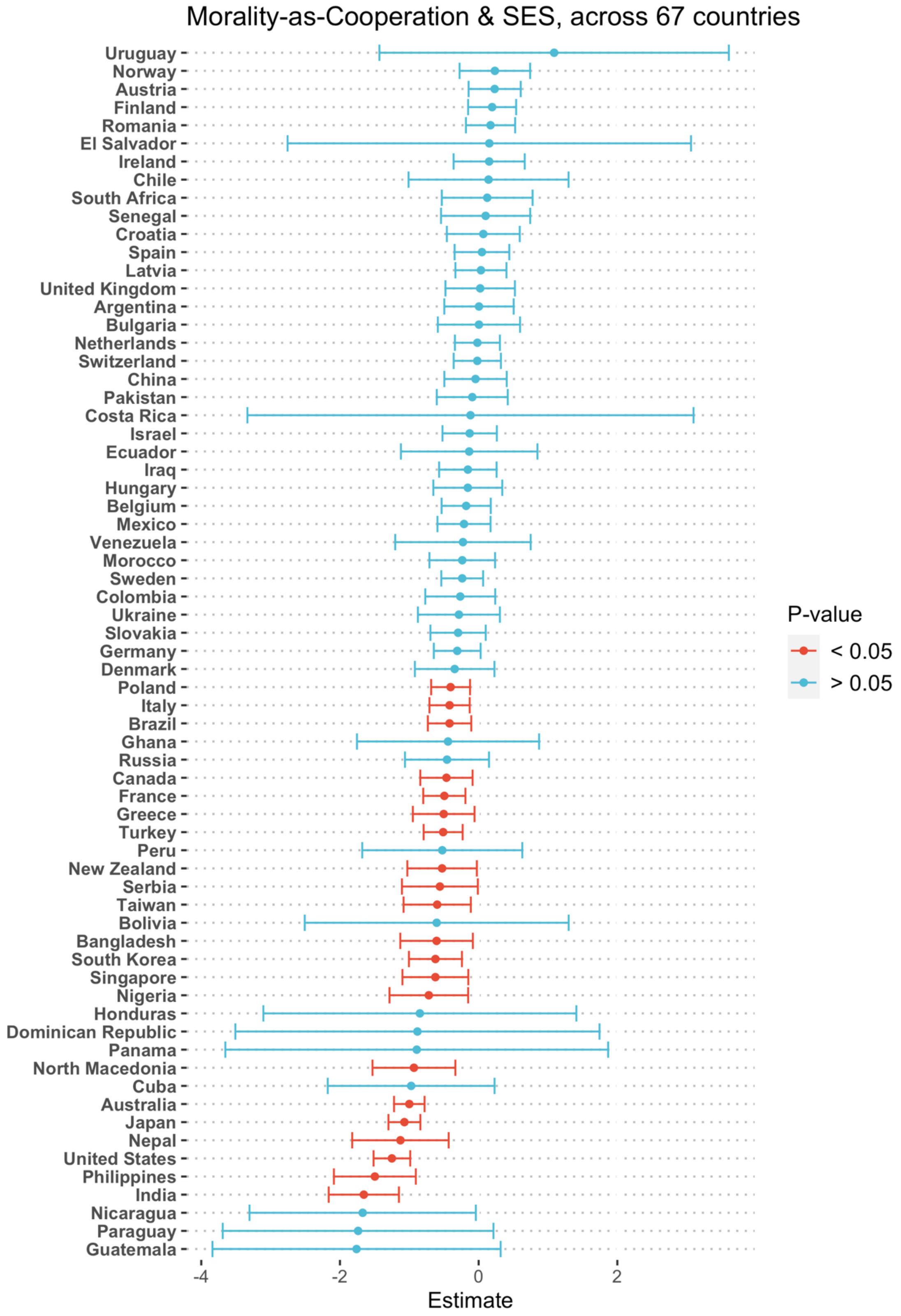

Fig. S4 | Nested OLS regression estimates for associations between Morality-as-Cooperation and Subjective Socioeconomic Status (SES) across all 67 countries. Dots indicate OLS regression estimates and error bars indicate 95% confidence intervals. Dots and errors bars in red indicate regression estimates with p-values < 0.05. Dots and error bars in light blue indicate regression estimates with p-values > 0.05. Ordinary Least Squares regressions (two-sided) for each of the 67 countries (i.e., Nested OLS). N = 50,563 survey participants.

3.3 Moral Circle

3.3.1. Table S13

Table S13 | Nested OLS regression results

Country	*DV*	*IV*	*Estimate*	*SE*	*t*	*df*	*p*
Argentina	MC	SES	-0.040	0.117	-0.342	719	0.732
Australia	MC	SES	0.069	0.057	1.212	2122	0.226
Austria	MC	SES	0.143	0.075	1.903	1586	0.057
Bangladesh	MC	SES	0.450	0.130	3.453	586	0.001
Belgium	MC	SES	0.194	0.095	2.040	1150	0.042
Bolivia	MC	SES	0.266	0.479	0.556	27	0.583
Brazil	MC	SES	-0.011	0.069	-0.157	2211	0.875
Bulgaria	MC	SES	-0.098	0.135	-0.721	655	0.471
Canada	MC	SES	0.096	0.093	1.034	954	0.302
Chile	MC	SES	0.419	0.279	1.502	95	0.137
China	MC	SES	0.066	0.075	0.881	1028	0.379
Colombia	MC	SES	0.031	0.099	0.313	1261	0.754
Costa Rica	MC	SES	0.695	0.824	0.843	23	0.408
Croatia	MC	SES	-0.175	0.141	-1.243	509	0.214
Cuba	MC	SES	0.228	0.392	0.582	41	0.563
Denmark	MC	SES	0.169	0.126	1.347	551	0.179
Dominican Republic	MC	SES	-0.445	0.498	-0.895	34	0.377
Ecuador	MC	SES	0.108	0.278	0.389	146	0.698
El Salvador	MC	SES	-0.349	0.732	-0.477	26	0.638
Finland	MC	SES	-0.127	0.079	-1.620	660	0.106
France	MC	SES	0.055	0.086	0.637	1113	0.524
Germany	MC	SES	-0.054	0.080	-0.674	1579	0.501
Ghana	MC	SES	-0.294	0.224	-1.309	388	0.192
Greece	MC	SES	0.073	0.132	0.550	634	0.583
Guatemala	MC	SES	0.411	0.441	0.932	46	0.356
Honduras	MC	SES	0.654	0.563	1.161	22	0.258
Hungary	MC	SES	0.210	0.136	1.541	504	0.124
India	MC	SES	0.042	0.092	0.453	722	0.650
Iraq	MC	SES	-0.038	0.097	-0.398	1126	0.691
Ireland	MC	SES	-0.062	0.108	-0.576	772	0.565
Israel	MC	SES	0.298	0.080	3.720	1243	0.000
Italy	MC	SES	0.091	0.081	1.123	1270	0.262
Japan	MC	SES	-0.069	0.068	-1.015	1228	0.310
Latvia	MC	SES	-0.017	0.098	-0.175	993	0.861
Mexico	MC	SES	-0.029	0.089	-0.325	1301	0.745
Morocco	MC	SES	0.088	0.137	0.638	793	0.524

Nepal	MC	SES	0.015	0.166	0.090	555	0.929
Netherlands	MC	SES	0.234	0.075	3.135	1295	0.002
New Zealand	MC	SES	0.083	0.117	0.713	507	0.476
Nicaragua	MC	SES	0.114	0.666	0.171	14	0.866
Nigeria	MC	SES	0.173	0.120	1.437	604	0.151
North Macedonia	MC	SES	-0.059	0.120	-0.490	713	0.624
Norway	MC	SES	0.148	0.113	1.310	524	0.191
Pakistan	MC	SES	-0.086	0.135	-0.638	554	0.524
Panama	MC	SES	-0.570	0.492	-1.158	16	0.264
Paraguay	MC	SES	0.067	0.673	0.100	14	0.922
Peru	MC	SES	0.066	0.313	0.212	89	0.833
Philippines	MC	SES	0.295	0.150	1.964	520	0.050
Poland	MC	SES	-0.077	0.073	-1.049	1803	0.294
Romania	MC	SES	-0.048	0.108	-0.447	998	0.655
Russia	MC	SES	-0.173	0.141	-1.230	505	0.219
Senegal	MC	SES	-0.235	0.126	-1.860	543	0.064
Serbia	MC	SES	-0.127	0.103	-1.236	1063	0.217
Singapore	MC	SES	0.017	0.135	0.127	558	0.899
Slovakia	MC	SES	0.263	0.100	2.621	1094	0.009
South Africa	MC	SES	-0.028	0.104	-0.269	925	0.788
South Korea	MC	SES	-0.210	0.088	-2.387	548	0.017
Spain	MC	SES	0.088	0.092	0.952	1088	0.341
Sweden	MC	SES	0.211	0.068	3.105	1566	0.002
Switzerland	MC	SES	0.162	0.091	1.785	1051	0.074
Taiwan	MC	SES	-0.095	0.089	-1.066	831	0.287
Turkey	MC	SES	0.042	0.069	0.611	1437	0.541
Ukraine	MC	SES	-0.087	0.143	-0.606	575	0.545
United Kingdom	MC	SES	-0.087	0.121	-0.718	544	0.473
United States	MC	SES	0.133	0.058	2.288	1469	0.022
Uruguay	MC	SES	0.430	0.607	0.709	47	0.482
Venezuela	MC	SES	-0.106	0.270	-0.395	94	0.694

Notes: MC = Moral Circle, SES = Subjective Socioeconomic Status. Model is a Nested Ordinary Least Square (OLS) regression (two-sided). Each row indicates a separate OLS regression.

3.3.2. Figure S5

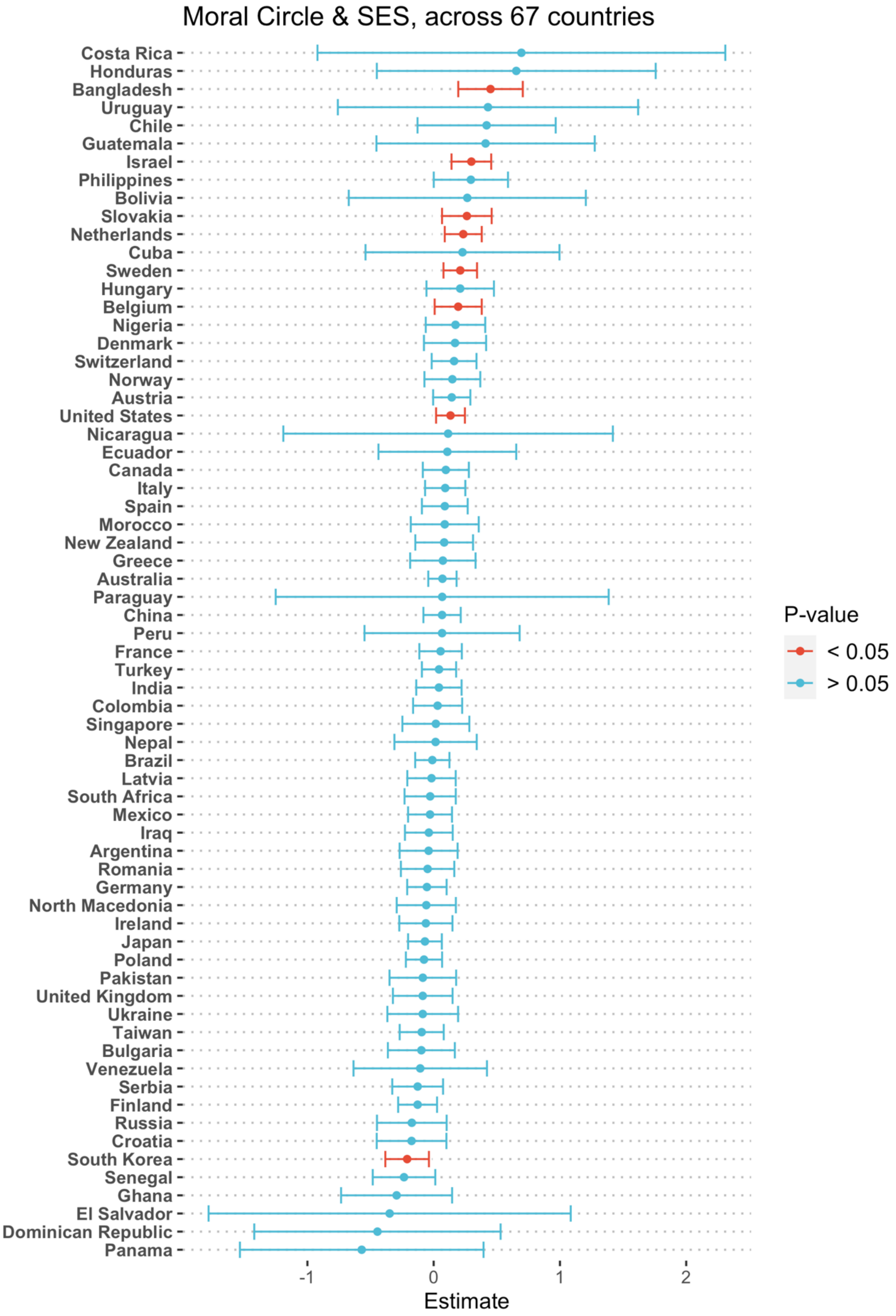

Fig. S5 | Nested OLS regression estimates for associations between the size of one's Moral Circle and Subjective Socioeconomic Status (SES) across all 67 countries. Dots indicate OLS regression estimates and error bars indicate 95% confidence intervals. Dots and errors bars in red indicate regression estimates with p-values < 0.05. Dots and error bars in light blue indicate regression estimates with p-values > 0.05. Ordinary Least Squares regressions (two-sided) for each of the 67 countries (i.e., Nested OLS). $N = 50{,}563$ survey participants.

3.4. Prosocial Intention

3.3.1. Table S14

Table S14 | Nested OLS regression results

Country	*DV*	*IV*	*Estimate*	*SE*	*t*	*df*	*p*
Argentina	PI	SES	-0.700	0.969	-0.723	719	0.470
Australia	PI	SES	-2.211	0.347	-6.362	2122	0.000
Austria	PI	SES	-2.317	0.575	-4.028	1586	0.000
Bangladesh	PI	SES	2.553	0.664	3.843	586	0.000
Belgium	PI	SES	-1.312	0.692	-1.895	1150	0.058
Bolivia	PI	SES	-5.179	5.400	-0.959	27	0.356
Brazil	PI	SES	-1.924	0.419	-4.592	2211	0.000
Bulgaria	PI	SES	0.918	0.465	1.973	655	0.049
Canada	PI	SES	-1.100	0.640	-1.720	954	0.086
Chile	PI	SES	-1.758	2.591	-0.679	95	0.500
China	PI	SES	-1.162	0.730	-1.591	1028	0.112
Colombia	PI	SES	-3.159	0.725	-4.355	1261	0.000
Costa Rica	PI	SES	-2.460	5.665	-0.434	23	0.670
Croatia	PI	SES	-1.795	0.905	-1.983	509	0.048
Cuba	PI	SES	0.947	5.161	0.184	41	0.857
Denmark	PI	SES	-0.709	0.766	-0.926	551	0.355
Dominican Republic	PI	SES	-9.087	3.866	-2.350	34	0.029
Ecuador	PI	SES	-3.640	2.019	-1.803	146	0.075
El Salvador	PI	SES	-4.859	5.267	-0.923	26	0.370
Finland	PI	SES	-4.012	0.776	-5.170	660	0.000
France	PI	SES	-2.668	0.563	-4.739	1113	0.000
Germany	PI	SES	0.599	0.332	1.802	1579	0.072
Ghana	PI	SES	-0.472	0.778	-0.606	388	0.545
Greece	PI	SES	-0.335	0.873	-0.384	634	0.701
Guatemala	PI	SES	-3.909	4.022	-0.972	46	0.339
Honduras	PI	SES	-3.113	3.683	-0.845	22	0.409
Hungary	PI	SES	-2.302	0.962	-2.392	504	0.017
India	PI	SES	-4.124	0.629	-6.555	722	0.000
Iraq	PI	SES	-0.535	0.542	-0.986	1126	0.325
Ireland	PI	SES	-0.222	0.564	-0.394	772	0.694
Israel	PI	SES	-0.950	0.556	-1.709	1243	0.088
Italy	PI	SES	-0.243	0.469	-0.517	1270	0.605
Japan	PI	SES	-2.233	0.427	-5.230	1228	0.000
Latvia	PI	SES	-1.472	0.614	-2.398	993	0.017
Mexico	PI	SES	-1.409	0.620	-2.274	1301	0.023

Morocco	PI	SES	-2.553	0.712	-3.588	793	0.000
Nepal	PI	SES	1.139	0.943	1.207	555	0.228
Netherlands	PI	SES	-2.723	0.599	-4.549	1295	0.000
New Zealand	PI	SES	-0.833	0.772	-1.080	507	0.281
Nicaragua	PI	SES	-5.714	5.131	-1.114	14	0.291
Nigeria	PI	SES	-0.828	0.565	-1.466	604	0.143
North Macedonia	PI	SES	0.283	0.756	0.374	713	0.708
Norway	PI	SES	-2.391	1.004	-2.381	524	0.018
Pakistan	PI	SES	-0.007	0.749	-0.009	554	0.993
Panama	PI	SES	-11.036	6.828	-1.616	16	0.140
Paraguay	PI	SES	-1.205	5.418	-0.222	14	0.829
Peru	PI	SES	0.131	3.079	0.043	89	0.966
Philippines	PI	SES	-0.748	0.744	-1.005	520	0.316
Poland	PI	SES	-1.048	0.429	-2.441	1803	0.015
Romania	PI	SES	0.462	0.609	0.758	998	0.448
Russia	PI	SES	-1.660	0.965	-1.720	505	0.086
Senegal	PI	SES	0.050	0.772	0.065	543	0.948
Serbia	PI	SES	-0.377	0.837	-0.450	1063	0.653
Singapore	PI	SES	-1.703	0.818	-2.083	558	0.038
Slovakia	PI	SES	-0.771	0.670	-1.150	1094	0.250
South Africa	PI	SES	-2.823	1.018	-2.774	925	0.006
South Korea	PI	SES	-2.068	0.685	-3.018	548	0.003
Spain	PI	SES	-1.884	0.688	-2.738	1088	0.006
Sweden	PI	SES	-1.772	0.590	-3.003	1566	0.003
Switzerland	PI	SES	-3.370	0.533	-6.329	1051	0.000
Taiwan	PI	SES	-0.952	0.626	-1.521	831	0.129
Turkey	PI	SES	-2.019	0.383	-5.268	1437	0.000
Ukraine	PI	SES	-1.671	0.891	-1.875	575	0.061
United Kingdom	PI	SES	-2.621	0.836	-3.137	544	0.002
United States	PI	SES	-1.800	0.378	-4.756	1469	0.000
Uruguay	PI	SES	-1.384	5.582	-0.248	47	0.806
Venezuela	PI	SES	-2.558	2.360	-1.084	94	0.283

Notes: PI = Prosocial Intention, SES = Subjective Socioeconomic status. Model is a Nested Ordinary Least Square (OLS) regression (two-sided). Each row indicates a separate OLS regression.

3.3.2. Figure S6

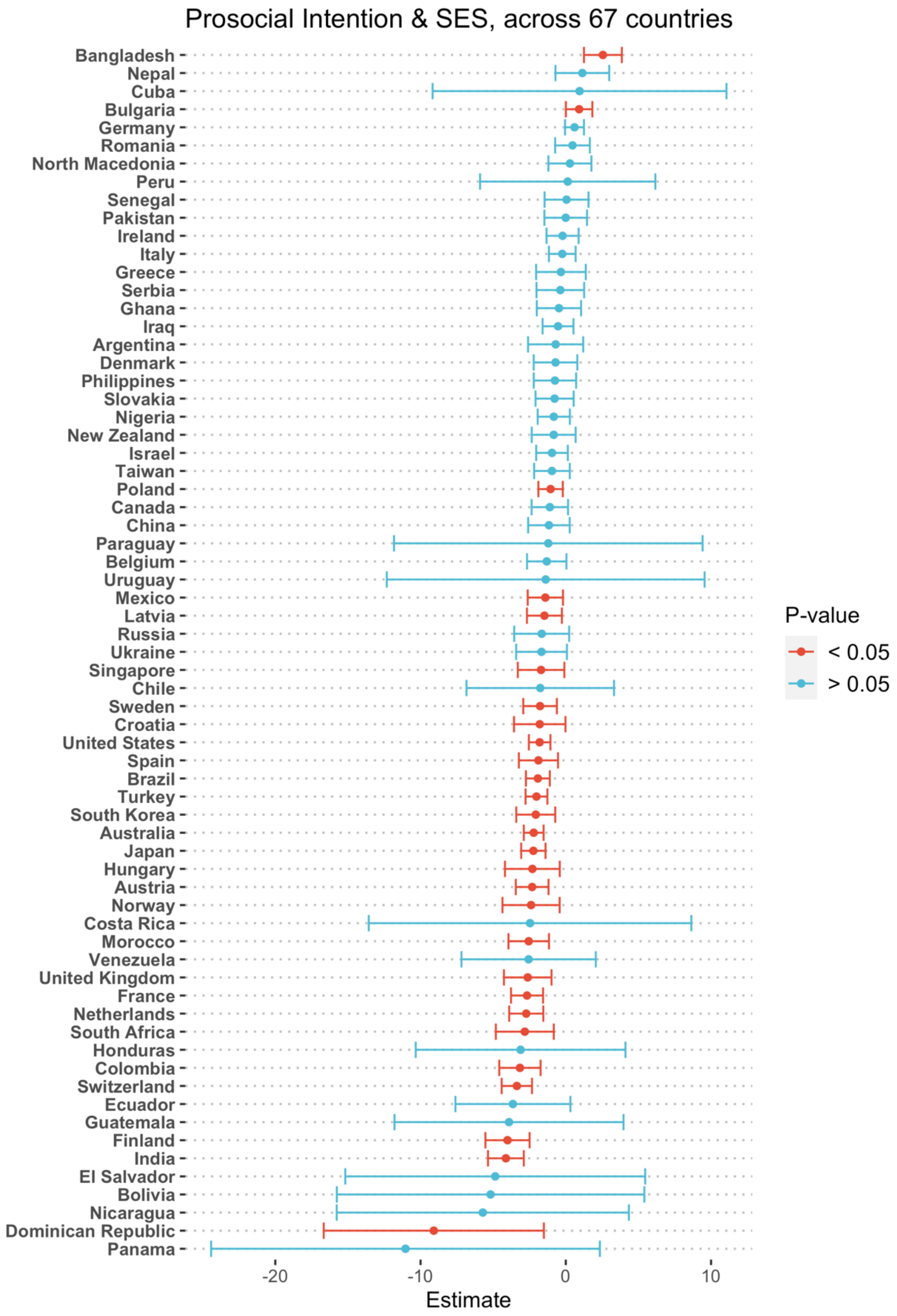

Fig. S6 | Nested OLS regression estimates for associations between Prosocial Intention (willingness to donate to a national and international charity) and Subjective Socioeconomic Status (SES) across all 67 countries. Dots indicate OLS regression estimates and error bars indicate 95% confidence intervals. Dots and errors bars in red indicate regression estimates with p-values < 0.05. Dots and error bars in light blue indicate regression estimates with p-values > 0.05. Ordinary Least Squares regressions (two-sided) for each of the 67 countries (i.e., Nested OLS). N = 50,563 survey participants.

4. Cross Validations

4.1. All models

4.1.1. Table S15

Table S15 | Cross validation results (10-folds, 200 repetitions)

	**Moral Identity**			**Morality-as-Cooperation**			**Moral Circle**			**Prosocial Intention**		
Model specification	*RMSE*	*MAE*	*AIC*	*RMSE*	*MAE*	*AIC*	*RMSE*	*MAE*	*AIC*	*RMSE*	*MAE*	*AIC*
~ 1	14.2	11.0	331202	11.6	8.92	315153	5.23	4.58	254064	35.6	30.1	416256
~ SES	14.1	10.9	329968	11.5	8.90	314314	5.23	4.57	253533	35.4	30.3	414020
~ GINI	14.1	10.8	330213	11.5	8.85	314739	5.22	4.56	253824	35.5	30.3	416116
~ SES + GINI	14.0	10.8	329075	11.5	8.83	313934	5.21	4.56	253298	35.3	30.3	413916
~ SES * GINI	14.0	10.8	329076	11.1	8.83	313925	5.21	4.56	253298	35.3	30.3	413917
~ SES + GINI + (1 | country)	13.4	10.4	325705	11.1	8.51	311377	5.12	4.45	251935	34.1	29.1	411078
~ SES * GINI + (1 | country)	13.4	10.4	325716	11.1	8.51	311379	5.12	4.45	251947	34.1	29.1	411086
~ SES + GINI + age + sex + (1 | country)	13.3	10.3	324769	11.1	8.50	310551	5.09	4.41	250890	33.9	29.0	409759
~ SES * GINI + age + sex + (1 | country)	13.3	10.3	324779	11.1	8.50	310554	5.09	4.41	250902	33.9	29.0	409768

Note: RMSE = Root Mean Square Error, MAE = Mean Absolute Error, AIC = Akaike Information Criterion

5. Multilevel Confirmatory Factor Analysis Alignment

Please note: For all supplementary results on the two Multilevel Confirmatory Factor Analysis Alignments, we were unable to export table output from the statistical environment R, due to characteristics of the packages used in these analysis. Therefore, the outcomes of these analysis are not reported as formatted tables, but instead as the direct output from the analyses. All analyses can be easily reproduced using the data and the script available at OSF.

5.1. Moral Identity

5.1.1. Table S16

Table S16 | Summary of results for factor loadings

Parameter tolerance value	1
Total number of items	670
Number of unique items parameters	67
Percentage of non-invariance parameters	8.5 %
R^2	.85

5.1.2. Table S17

Table S17 | Estimated Item Parameters for Factor Loadings

	moralid1	moralid2	moralid3	moralid4	moralid5	moralid6	moralid7	moralid8	moralid9	moralid10
Argentina	1.028	1.127	1.267	-0.317	1.614	1.581	-0.770	1.444	1.681	1.417
Australia	1.028	2.296	1.267	-0.317	1.614	1.581	-0.770	1.444	1.681	1.417
Austria	1.028	1.127	1.267	-0.317	1.614	1.581	-0.770	1.444	1.681	1.417
Bangladesh	2.977	3.106	1.267	-0.317	1.614	1.581	-2.178	1.444	1.681	2.930
Belgium	1.028	1.127	1.267	-0.317	1.614	1.581	-0.770	1.444	1.681	1.417
Bolivia	1.028	1.127	1.267	-0.317	0.554	1.581	-0.770	1.444	1.681	1.417
Brazil	1.028	1.127	1.267	-0.317	1.614	1.581	-0.770	1.444	1.681	1.417
Bulgaria	1.028	1.127	1.267	-0.317	1.614	1.581	-0.770	1.444	1.681	1.417
Canada	1.028	2.322	1.267	-1.334	1.614	1.581	-2.009	1.444	1.681	2.508
Chile	1.028	1.127	1.267	-0.317	1.614	1.581	-0.770	1.444	1.681	1.417
China	1.028	1.127	1.267	-0.317	1.614	1.581	-0.770	1.444	1.681	1.417
Colombia	1.028	1.127	1.267	-0.317	1.614	1.581	-0.770	1.444	1.681	1.417
Costa Rica	1.028	1.127	1.267	-0.317	1.614	1.581	-0.770	0.214	0.529	1.417
Croatia	1.028	1.127	1.267	-0.317	1.614	1.581	-0.770	1.444	1.681	1.417
Cuba	1.028	1.127	1.267	-0.317	1.614	1.581	-0.770	1.444	1.681	1.417
Denmark	2.300	2.371	1.267	-0.317	1.614	1.581	-0.770	1.444	1.681	2.533
Dominican Republic	1.028	1.127	1.267	-0.317	1.614	1.581	-0.770	1.444	1.681	1.417
Ecuador	1.028	1.127	1.267	-0.317	1.614	1.581	-0.770	1.444	1.681	1.417
El Salvador	1.028	1.127	1.267	-0.317	1.614	1.581	-0.770	1.444	1.681	1.417
Finland	1.028	1.127	1.267	-0.317	1.614	1.581	-2.124	1.444	1.681	1.417
France	1.028	1.127	1.267	0.947	1.614	1.581	0.388	1.444	1.681	1.417
Germany	2.064	2.271	1.267	-0.317	1.614	1.581	-0.770	1.444	1.681	1.417
Ghana	1.028	1.127	1.267	-0.317	1.614	1.581	0.588	1.444	1.681	1.417
Greece	1.028	1.127	1.267	-0.317	1.614	1.581	-2.448	1.444	1.681	2.885
Guatemala	2.675	2.318	1.267	-1.377	1.614	1.581	-0.770	1.444	1.681	1.417
Honduras	1.028	1.127	1.267	-0.317	1.614	1.581	-0.770	1.444	0.678	1.417
Hungary	1.028	1.127	1.267	-0.317	1.614	1.581	-0.770	1.444	1.681	1.417
India	1.028	1.127	-0.449	-0.317	-0.214	-0.210	-1.992	0.140	0.499	1.417
Iraq	1.028	1.127	1.267	-0.317	1.614	1.581	-0.770	1.444	1.681	1.417
Ireland	1.028	1.127	1.267	-0.317	1.614	1.581	-0.770	1.444	1.681	1.417
Israel	1.028	1.127	1.267	-0.317	1.614	1.581	-2.045	1.444	1.681	1.417
Italy	1.028	1.127	1.267	-0.317	1.614	1.581	-0.770	1.444	1.681	1.417
Japan	1.028	1.127	1.267	0.775	1.614	1.581	-0.770	1.444	1.681	1.417
Latvia	1.028	1.127	1.267	-0.317	1.614	1.581	-0.770	1.444	1.681	1.417
Mexico	1.028	1.127	1.267	-0.317	1.614	1.581	-0.770	1.444	1.681	1.417
Morocco	1.028	1.127	1.267	-0.317	1.614	1.581	-0.770	1.444	1.681	1.417
Nepal	1.028	1.127	1.267	-0.317	1.614	1.581	-0.770	1.444	1.681	1.417
Netherlands	1.028	1.127	1.267	-0.317	1.614	1.581	-0.770	1.444	1.681	1.417
New Zealand	1.028	2.319	1.267	-1.513	1.614	1.581	-2.340	1.444	1.681	2.572
Nicaragua	2.569	1.127	1.267	-0.317	1.614	1.581	-0.770	1.444	0.596	1.417
Nigeria	1.028	1.127	1.267	-0.317	1.614	1.581	-0.770	1.444	1.681	1.417
North Macedonia	1.028	1.127	1.267	-0.317	1.614	1.581	-0.770	1.444	1.681	1.417
Norway	1.028	1.127	1.267	-0.317	1.614	1.581	-0.770	1.444	1.681	1.417
Pakistan	2.702	2.615	1.267	-2.381	1.614	1.581	-2.542	1.444	1.681	2.725
Panama	1.028	1.127	1.267	-0.317	1.614	1.581	-0.770	1.444	1.681	1.417
Paraguay	1.028	1.127	1.267	-0.317	1.614	0.545	-0.770	1.444	1.681	1.417
Peru	1.028	1.127	1.267	-0.317	1.614	1.581	-0.770	1.444	1.681	1.417
Philippines	1.028	1.127	1.267	-0.317	1.614	1.581	-0.770	1.444	1.681	1.417
Poland	2.038	2.198	1.267	-0.317	1.614	1.581	-0.770	1.444	1.681	1.417
Romania	1.028	1.127	1.267	-0.317	1.614	1.581	-0.770	1.444	1.681	1.417
Russia	2.388	2.830	1.267	-0.317	1.614	1.581	-0.770	1.444	1.681	3.067
Senegal	1.028	1.127	1.267	-0.317	1.614	1.581	-0.770	1.444	1.681	1.417
Serbia	1.028	1.127	1.267	-0.317	1.614	1.581	-0.770	1.444	1.681	1.417
Singapore	1.028	1.127	1.267	-0.317	1.614	1.581	-0.770	1.444	1.681	1.417
Slovakia	1.028	1.127	1.267	-0.317	1.614	1.581	-0.770	1.444	1.681	1.417
South Africa	1.028	1.127	1.267	-0.317	1.614	1.581	-0.770	1.444	1.681	1.417
South Korea	1.028	1.127	1.267	-0.317	1.614	1.581	-0.770	1.444	1.681	1.417
Spain	1.028	1.127	1.267	-0.317	1.614	1.581	-0.770	1.444	1.681	1.417
Sweden	1.028	1.127	1.267	-0.317	1.614	1.581	-0.770	1.444	1.681	1.417
Switzerland	1.028	1.127	1.267	-0.317	1.614	1.581	-0.770	1.444	1.681	1.417
Taiwan	1.028	1.127	1.267	0.761	1.614	1.581	0.247	1.444	1.681	1.417
Turkey	1.028	1.127	1.267	-0.317	1.614	1.581	0.326	1.444	1.681	1.417
Ukraine	1.028	1.127	1.267	-0.317	1.614	1.581	-0.770	1.444	1.681	1.417
United Kingdom	1.028	1.127	1.267	-0.317	1.614	1.581	-0.770	1.444	1.681	1.417
United States	1.028	1.127	1.267	0.977	1.614	1.581	0.698	1.444	1.681	1.417
Uruguay	1.028	1.127	1.267	-0.317	1.614	1.581	-0.770	1.444	1.681	1.417
Venezuela	1.028	1.127	1.267	-0.317	1.614	1.581	-0.770	1.444	1.681	1.417

5.1.3. Table S18

Table S18 | Estimated DIF Effects for Factor Loadings

	moralid1	moralid2	moralid3	moralid4	moralid5	moralid6	moralid7	moralid8	moralid9	moralid10
Argentina	0.000	0.000	0.000	0.000	0.000	0.000	0.000	0.000	0.000	0.000
Australia	0.000	1.169	0.000	0.000	0.000	0.000	0.000	0.000	0.000	0.000
Austria	0.000	0.000	0.000	0.000	0.000	0.000	0.000	0.000	0.000	0.000
Bangladesh	1.949	1.979	0.000	0.000	0.000	0.000	-1.407	0.000	0.000	1.513
Belgium	0.000	0.000	0.000	0.000	0.000	0.000	0.000	0.000	0.000	0.000
Bolivia	0.000	0.000	0.000	0.000	-1.060	0.000	0.000	0.000	0.000	0.000
Brazil	0.000	0.000	0.000	0.000	0.000	0.000	0.000	0.000	0.000	0.000
Bulgaria	0.000	0.000	0.000	0.000	0.000	0.000	0.000	0.000	0.000	0.000
Canada	0.000	1.195	0.000	-1.017	0.000	0.000	-1.238	0.000	0.000	1.091
Chile	0.000	0.000	0.000	0.000	0.000	0.000	0.000	0.000	0.000	0.000
China	0.000	0.000	0.000	0.000	0.000	0.000	0.000	0.000	0.000	0.000
Colombia	0.000	0.000	0.000	0.000	0.000	0.000	0.000	0.000	0.000	0.000
Costa Rica	0.000	0.000	0.000	0.000	0.000	0.000	0.000	-1.230	-1.152	0.000
Croatia	0.000	0.000	0.000	0.000	0.000	0.000	0.000	0.000	0.000	0.000
Cuba	0.000	0.000	0.000	0.000	0.000	0.000	0.000	0.000	0.000	0.000
Denmark	1.272	1.244	0.000	0.000	0.000	0.000	0.000	0.000	0.000	1.116
Dominican Republic	0.000	0.000	0.000	0.000	0.000	0.000	0.000	0.000	0.000	0.000
Ecuador	0.000	0.000	0.000	0.000	0.000	0.000	0.000	0.000	0.000	0.000
El Salvador	0.000	0.000	0.000	0.000	0.000	0.000	0.000	0.000	0.000	0.000
Finland	0.000	0.000	0.000	0.000	0.000	0.000	-1.354	0.000	0.000	0.000
France	0.000	0.000	0.000	1.264	0.000	0.000	1.158	0.000	0.000	0.000
Germany	1.036	1.144	0.000	0.000	0.000	0.000	0.000	0.000	0.000	0.000
Ghana	0.000	0.000	0.000	0.000	0.000	0.000	1.358	0.000	0.000	0.000
Greece	0.000	0.000	0.000	0.000	0.000	0.000	-1.678	0.000	0.000	1.468
Guatemala	1.647	1.190	0.000	-1.060	0.000	0.000	0.000	0.000	0.000	0.000
Honduras	0.000	0.000	0.000	0.000	0.000	0.000	0.000	0.000	-1.003	0.000
Hungary	0.000	0.000	0.000	0.000	0.000	0.000	0.000	0.000	0.000	0.000
India	0.000	0.000	-1.716	0.000	-1.827	-1.792	-1.221	-1.305	-1.182	0.000
Iraq	0.000	0.000	0.000	0.000	0.000	0.000	0.000	0.000	0.000	0.000
Ireland	0.000	0.000	0.000	0.000	0.000	0.000	0.000	0.000	0.000	0.000
Israel	0.000	0.000	0.000	0.000	0.000	0.000	-1.275	0.000	0.000	0.000
Italy	0.000	0.000	0.000	0.000	0.000	0.000	0.000	0.000	0.000	0.000
Japan	0.000	0.000	0.000	1.092	0.000	0.000	0.000	0.000	0.000	0.000
Latvia	0.000	0.000	0.000	0.000	0.000	0.000	0.000	0.000	0.000	0.000
Mexico	0.000	0.000	0.000	0.000	0.000	0.000	0.000	0.000	0.000	0.000
Morocco	0.000	0.000	0.000	0.000	0.000	0.000	0.000	0.000	0.000	0.000
Nepal	0.000	0.000	0.000	0.000	0.000	0.000	0.000	0.000	0.000	0.000
Netherlands	0.000	0.000	0.000	0.000	0.000	0.000	0.000	0.000	0.000	0.000
New Zealand	0.000	1.192	0.000	-1.196	0.000	0.000	-1.569	0.000	0.000	1.155
Nicaragua	1.541	0.000	0.000	0.000	0.000	0.000	0.000	0.000	-1.085	0.000
Nigeria	0.000	0.000	0.000	0.000	0.000	0.000	0.000	0.000	0.000	0.000
North Macedonia	0.000	0.000	0.000	0.000	0.000	0.000	0.000	0.000	0.000	0.000
Norway	0.000	0.000	0.000	0.000	0.000	0.000	0.000	0.000	0.000	0.000
Pakistan	1.674	1.487	0.000	-2.064	0.000	0.000	-1.772	0.000	0.000	1.308
Panama	0.000	0.000	0.000	0.000	0.000	0.000	0.000	0.000	0.000	0.000
Paraguay	0.000	0.000	0.000	0.000	0.000	-1.036	0.000	0.000	0.000	0.000
Peru	0.000	0.000	0.000	0.000	0.000	0.000	0.000	0.000	0.000	0.000
Philippines	0.000	0.000	0.000	0.000	0.000	0.000	0.000	0.000	0.000	0.000
Poland	1.009	1.071	0.000	0.000	0.000	0.000	0.000	0.000	0.000	0.000
Romania	0.000	0.000	0.000	0.000	0.000	0.000	0.000	0.000	0.000	0.000
Russia	1.359	1.703	0.000	0.000	0.000	0.000	0.000	0.000	0.000	1.650
Senegal	0.000	0.000	0.000	0.000	0.000	0.000	0.000	0.000	0.000	0.000
Serbia	0.000	0.000	0.000	0.000	0.000	0.000	0.000	0.000	0.000	0.000
Singapore	0.000	0.000	0.000	0.000	0.000	0.000	0.000	0.000	0.000	0.000
Slovakia	0.000	0.000	0.000	0.000	0.000	0.000	0.000	0.000	0.000	0.000
South Africa	0.000	0.000	0.000	0.000	0.000	0.000	0.000	0.000	0.000	0.000
South Korea	0.000	0.000	0.000	0.000	0.000	0.000	0.000	0.000	0.000	0.000
Spain	0.000	0.000	0.000	0.000	0.000	0.000	0.000	0.000	0.000	0.000
Sweden	0.000	0.000	0.000	0.000	0.000	0.000	0.000	0.000	0.000	0.000
Switzerland	0.000	0.000	0.000	0.000	0.000	0.000	0.000	0.000	0.000	0.000
Taiwan	0.000	0.000	0.000	1.078	0.000	0.000	1.017	0.000	0.000	0.000
Turkey	0.000	0.000	0.000	0.000	0.000	0.000	1.096	0.000	0.000	0.000
Ukraine	0.000	0.000	0.000	0.000	0.000	0.000	0.000	0.000	0.000	0.000
United Kingdom	0.000	0.000	0.000	0.000	0.000	0.000	0.000	0.000	0.000	0.000
United States	0.000	0.000	0.000	1.294	0.000	0.000	1.468	0.000	0.000	0.000
Uruguay	0.000	0.000	0.000	0.000	0.000	0.000	0.000	0.000	0.000	0.000
Venezuela	0.000	0.000	0.000	0.000	0.000	0.000	0.000	0.000	0.000	0.000

5.1.4. Table S19

Table S19 | Summary of results for factor intercepts

Parameter tolerance value	1
Total number of items	670
Number of unique items parameters	62
Percentage of non-invariance parameters	7.8 %
R^2	.99

5.1.5. Table S20

Table S20 | Estimated Item Parameters for Factor Intercepts

	moralid1	moralid2	moralid3	moralid4	moralid5	moralid6	moralid7	moralid8	moralid9	moralid10
Argentina	8.592	8.115	4.766	1.129	6.101	5.678	2.671	5.071	5.521	7.383
Australia	8.592	8.115	4.766	2.212	6.101	5.678	2.671	5.071	5.521	7.383
Austria	8.592	8.115	4.766	1.129	6.101	5.678	2.671	5.071	6.525	7.383
Bangladesh	8.592	8.115	4.766	2.467	6.101	5.678	2.671	5.071	5.521	9.005
Belgium	8.592	8.115	4.766	1.129	6.101	5.678	2.671	5.071	5.521	7.383
Bolivia	8.592	8.115	4.766	1.129	6.101	5.678	2.671	5.071	5.521	5.538
Brazil	8.592	8.115	4.766	1.129	6.101	5.678	2.671	5.071	5.521	7.383
Bulgaria	8.592	8.115	4.766	1.129	6.101	5.678	2.671	5.071	5.521	7.383
Canada	8.592	8.115	4.766	1.129	6.101	5.678	2.671	5.071	5.521	7.383
Chile	8.592	8.115	4.766	1.129	6.101	5.678	2.671	5.071	5.521	6.037
China	8.592	8.115	4.766	2.588	6.101	5.678	2.671	5.071	5.521	7.383
Colombia	8.592	8.115	4.766	1.129	6.101	5.678	2.671	5.071	5.521	7.383
Costa Rica	8.592	8.115	4.766	1.129	6.101	5.678	2.671	5.071	5.521	5.622
Croatia	8.592	8.115	4.766	1.129	6.101	5.678	2.671	5.071	5.521	7.383
Cuba	8.592	9.131	4.766	1.129	6.101	5.678	2.671	5.071	5.521	7.383
Denmark	8.592	8.115	4.766	1.129	6.101	5.678	2.671	5.071	5.521	7.383
Dominican Republic	8.592	8.115	4.766	1.129	6.101	5.678	2.671	6.195	5.521	7.383
Ecuador	8.592	8.115	4.766	1.129	6.101	5.678	2.671	5.071	5.521	6.175
El Salvador	8.592	8.115	4.766	1.129	6.101	5.678	2.671	5.071	4.278	7.383
Finland	8.592	8.115	3.306	1.129	6.101	5.678	1.507	5.071	5.521	7.383
France	8.592	8.115	4.766	2.798	6.101	5.678	4.079	5.071	5.521	7.383
Germany	8.592	8.115	3.678	1.129	6.101	5.678	2.671	5.071	5.521	7.383
Ghana	4.723	5.419	4.766	1.129	6.101	5.678	2.671	5.071	6.824	5.152
Greece	8.592	8.115	4.766	1.129	6.101	5.678	2.671	3.988	5.521	7.383
Guatemala	8.592	8.115	4.766	1.129	6.101	5.678	2.671	5.071	5.521	7.383
Honduras	8.592	8.115	4.766	1.129	6.101	5.678	2.671	5.071	6.622	7.383
Hungary	8.592	8.115	4.766	1.129	6.101	5.678	2.671	5.071	5.521	7.383
India	8.592	8.115	4.766	1.129	6.101	5.678	2.671	5.071	5.521	7.383
Iraq	8.592	8.115	4.766	2.752	6.101	5.678	2.671	4.062	5.521	7.383
Ireland	8.592	8.115	4.766	1.129	6.101	5.678	2.671	5.071	5.521	7.383
Israel	8.592	8.115	4.766	1.129	6.101	5.678	2.671	5.071	5.521	7.383
Italy	8.592	8.115	4.766	1.129	6.101	5.678	2.671	5.071	5.521	7.383
Japan	7.181	6.813	4.766	3.982	6.101	5.678	4.226	5.071	5.521	7.383
Latvia	8.592	8.115	4.766	1.129	6.101	5.678	2.671	5.071	5.521	7.383
Mexico	8.592	8.115	4.766	1.129	6.101	5.678	2.671	5.071	5.521	7.383
Morocco	8.592	8.115	4.766	1.129	6.101	5.678	2.671	5.071	5.521	7.383
Nepal	8.592	8.115	4.766	1.129	6.101	5.678	2.671	5.071	6.805	7.383
Netherlands	8.592	8.115	4.766	1.129	6.101	5.678	2.671	5.071	5.521	7.383
New Zealand	8.592	8.115	4.766	1.129	6.101	5.678	2.671	5.071	5.521	7.383
Nicaragua	8.592	8.115	4.766	1.129	6.101	5.678	2.671	5.071	5.521	7.383
Nigeria	8.592	8.115	4.766	1.129	6.101	5.678	2.671	5.071	5.521	7.383
North Macedonia	8.592	8.115	4.766	1.129	6.101	5.678	2.671	5.071	5.521	7.383
Norway	8.592	8.115	4.766	1.129	6.101	5.678	2.671	5.071	5.521	7.383
Pakistan	8.592	8.115	4.766	1.129	6.101	5.678	2.671	5.071	5.521	7.383
Panama	8.592	9.281	4.766	1.129	6.101	5.678	2.671	5.071	5.521	7.383
Paraguay	8.592	8.115	4.766	1.129	6.101	5.678	2.671	5.071	4.306	5.364
Peru	8.592	8.115	4.766	1.129	6.101	5.678	2.671	5.071	5.521	7.383
Philippines	8.592	8.115	4.766	2.750	6.101	5.678	2.671	5.071	5.521	7.383
Poland	8.592	8.115	4.766	1.129	6.101	5.678	3.862	5.071	5.521	7.383
Romania	8.592	8.115	4.766	1.129	6.101	5.678	2.671	5.071	5.521	7.383
Russia	8.592	8.115	4.766	1.129	6.101	5.678	3.695	3.818	3.328	7.383
Senegal	8.592	8.115	4.766	1.129	6.101	5.678	2.671	5.071	5.521	7.383
Serbia	8.592	8.115	4.766	1.129	6.101	5.678	2.671	3.525	5.521	7.383
Singapore	8.592	8.115	4.766	2.480	6.101	5.678	2.671	5.071	5.521	7.383
Slovakia	8.592	8.115	4.766	1.129	6.101	5.678	4.021	5.071	5.521	7.383
South Africa	8.592	8.115	4.766	1.129	6.101	5.678	2.671	5.071	5.521	7.383
South Korea	7.363	6.779	5.905	3.630	6.101	5.678	4.242	5.071	5.521	7.383
Spain	8.592	8.115	4.766	1.129	6.101	5.678	2.671	5.071	5.521	7.383
Sweden	8.592	8.115	4.766	1.129	6.101	5.678	2.671	5.071	5.521	7.383
Switzerland	8.592	8.115	4.766	1.129	6.101	5.678	2.671	5.071	5.521	7.383
Taiwan	8.592	8.115	4.766	3.085	6.101	5.678	4.010	5.071	5.521	6.212
Turkey	8.592	8.115	4.766	1.129	6.101	5.678	2.671	5.071	5.521	7.383
Ukraine	8.592	8.115	4.766	1.129	6.101	5.678	2.671	5.071	5.521	7.383
United Kingdom	8.592	8.115	4.766	1.129	6.101	5.678	2.671	5.071	5.521	8.397
United States	8.592	8.115	4.766	2.742	6.101	5.678	2.671	5.071	5.521	7.383
Uruguay	8.592	8.115	4.766	1.129	6.101	5.678	2.671	5.071	5.521	7.383
Venezuela	8.592	8.115	4.766	1.129	6.101	5.678	2.671	5.071	5.521	5.893

5.1.6. Table S21

Table S21 | Estimated DIF Effects for Factor Intercepts

	moralid1	moralid2	moralid3	moralid4	moralid5	moralid6	moralid7	moralid8	moralid9	moralid10
Argentina	0.000	0.000	0.000	0.000	0	0	0.000	0.000	0.000	0.000
Australia	0.000	0.000	0.000	1.083	0	0	0.000	0.000	0.000	0.000
Austria	0.000	0.000	0.000	0.000	0	0	0.000	0.000	1.003	0.000
Bangladesh	0.000	0.000	0.000	1.338	0	0	0.000	0.000	0.000	1.621
Belgium	0.000	0.000	0.000	0.000	0	0	0.000	0.000	0.000	0.000
Bolivia	0.000	0.000	0.000	0.000	0	0	0.000	0.000	0.000	-1.845
Brazil	0.000	0.000	0.000	0.000	0	0	0.000	0.000	0.000	0.000
Bulgaria	0.000	0.000	0.000	0.000	0	0	0.000	0.000	0.000	0.000
Canada	0.000	0.000	0.000	0.000	0	0	0.000	0.000	0.000	0.000
Chile	0.000	0.000	0.000	0.000	0	0	0.000	0.000	0.000	-1.347
China	0.000	0.000	0.000	1.459	0	0	0.000	0.000	0.000	0.000
Colombia	0.000	0.000	0.000	0.000	0	0	0.000	0.000	0.000	0.000
Costa Rica	0.000	0.000	0.000	0.000	0	0	0.000	0.000	0.000	-1.762
Croatia	0.000	0.000	0.000	0.000	0	0	0.000	0.000	0.000	0.000
Cuba	0.000	1.016	0.000	0.000	0	0	0.000	0.000	0.000	0.000
Denmark	0.000	0.000	0.000	0.000	0	0	0.000	0.000	0.000	0.000
Dominican Republic	0.000	0.000	0.000	0.000	0	0	0.000	1.123	0.000	0.000
Ecuador	0.000	0.000	0.000	0.000	0	0	0.000	0.000	0.000	-1.208
El Salvador	0.000	0.000	0.000	0.000	0	0	0.000	0.000	-1.244	0.000
Finland	0.000	0.000	-1.460	0.000	0	0	-1.164	0.000	0.000	0.000
France	0.000	0.000	0.000	1.669	0	0	1.408	0.000	0.000	0.000
Germany	0.000	0.000	-1.089	0.000	0	0	0.000	0.000	0.000	0.000
Ghana	-3.869	-2.697	0.000	0.000	0	0	0.000	0.000	1.303	-2.231
Greece	0.000	0.000	0.000	0.000	0	0	0.000	-1.083	0.000	0.000
Guatemala	0.000	0.000	0.000	0.000	0	0	0.000	0.000	0.000	0.000
Honduras	0.000	0.000	0.000	0.000	0	0	0.000	0.000	1.101	0.000
Hungary	0.000	0.000	0.000	0.000	0	0	0.000	0.000	0.000	0.000
India	0.000	0.000	0.000	0.000	0	0	0.000	0.000	0.000	0.000
Iraq	0.000	0.000	0.000	1.623	0	0	0.000	-1.010	0.000	0.000
Ireland	0.000	0.000	0.000	0.000	0	0	0.000	0.000	0.000	0.000
Israel	0.000	0.000	0.000	0.000	0	0	0.000	0.000	0.000	0.000
Italy	0.000	0.000	0.000	0.000	0	0	0.000	0.000	0.000	0.000
Japan	-1.411	-1.302	0.000	2.853	0	0	1.556	0.000	0.000	0.000
Latvia	0.000	0.000	0.000	0.000	0	0	0.000	0.000	0.000	0.000
Mexico	0.000	0.000	0.000	0.000	0	0	0.000	0.000	0.000	0.000
Morocco	0.000	0.000	0.000	0.000	0	0	0.000	0.000	0.000	0.000
Nepal	0.000	0.000	0.000	0.000	0	0	0.000	0.000	1.284	0.000
Netherlands	0.000	0.000	0.000	0.000	0	0	0.000	0.000	0.000	0.000
New Zealand	0.000	0.000	0.000	0.000	0	0	0.000	0.000	0.000	0.000
Nicaragua	0.000	0.000	0.000	0.000	0	0	0.000	0.000	0.000	0.000
Nigeria	0.000	0.000	0.000	0.000	0	0	0.000	0.000	0.000	0.000
North Macedonia	0.000	0.000	0.000	0.000	0	0	0.000	0.000	0.000	0.000
Norway	0.000	0.000	0.000	0.000	0	0	0.000	0.000	0.000	0.000
Pakistan	0.000	0.000	0.000	0.000	0	0	0.000	0.000	0.000	0.000
Panama	0.000	1.166	0.000	0.000	0	0	0.000	0.000	0.000	0.000
Paraguay	0.000	0.000	0.000	0.000	0	0	0.000	0.000	-1.215	-2.019
Peru	0.000	0.000	0.000	0.000	0	0	0.000	0.000	0.000	0.000
Philippines	0.000	0.000	0.000	1.621	0	0	0.000	0.000	0.000	0.000
Poland	0.000	0.000	0.000	0.000	0	0	1.191	0.000	0.000	0.000
Romania	0.000	0.000	0.000	0.000	0	0	0.000	0.000	0.000	0.000
Russia	0.000	0.000	0.000	0.000	0	0	1.025	-1.253	-2.194	0.000
Senegal	0.000	0.000	0.000	0.000	0	0	0.000	0.000	0.000	0.000
Serbia	0.000	0.000	0.000	0.000	0	0	0.000	-1.547	0.000	0.000
Singapore	0.000	0.000	0.000	1.351	0	0	0.000	0.000	0.000	0.000
Slovakia	0.000	0.000	0.000	0.000	0	0	1.350	0.000	0.000	0.000
South Africa	0.000	0.000	0.000	0.000	0	0	0.000	0.000	0.000	0.000
South Korea	-1.229	-1.336	1.138	2.501	0	0	1.571	0.000	0.000	0.000
Spain	0.000	0.000	0.000	0.000	0	0	0.000	0.000	0.000	0.000
Sweden	0.000	0.000	0.000	0.000	0	0	0.000	0.000	0.000	0.000
Switzerland	0.000	0.000	0.000	0.000	0	0	0.000	0.000	0.000	0.000
Taiwan	0.000	0.000	0.000	1.956	0	0	1.339	0.000	0.000	-1.172
Turkey	0.000	0.000	0.000	0.000	0	0	0.000	0.000	0.000	0.000
Ukraine	0.000	0.000	0.000	0.000	0	0	0.000	0.000	0.000	0.000
United Kingdom	0.000	0.000	0.000	0.000	0	0	0.000	0.000	0.000	1.013
United States	0.000	0.000	0.000	1.614	0	0	0.000	0.000	0.000	0.000
Uruguay	0.000	0.000	0.000	0.000	0	0	0.000	0.000	0.000	0.000
Venezuela	0.000	0.000	0.000	0.000	0	0	0.000	0.000	0.000	-1.490

5.2. Morality-as-Cooperation

5.2.1. Table S22

Table S22 | Summary of results for factor loadings

Parameter tolerance value	1
Total number of items	469
Number of unique items parameters	56
Percentage of non-invariance parameters	10.4 %
R^2	.62

5.2.2. Table S23

Table S23 | Estimated Item Parameters for Factor Loadings

	mac_1	mac_2	mac_3	mac_4	mac_5	mac_6	mac_7
Argentina	1.660	1.766	1.638	1.684	1.178	-0.501	-0.820
Australia	1.660	1.766	1.638	1.684	1.178	0.809	0.553
Austria	1.660	1.766	1.638	1.684	1.178	0.809	0.553
Bangladesh	1.660	1.766	1.638	1.684	-0.500	-1.029	-0.787
Belgium	1.660	1.766	1.638	1.684	1.178	0.809	0.553
Bolivia	1.660	1.766	1.638	1.684	1.178	-0.913	-1.147
Brazil	1.660	1.766	1.638	1.684	1.178	0.809	0.553
Bulgaria	1.660	1.766	1.638	1.684	1.178	0.809	0.553
Canada	1.660	1.766	1.638	1.684	1.178	0.809	0.553
Chile	1.660	1.766	1.638	1.684	-0.195	0.809	0.553
China	1.660	1.766	1.638	1.684	1.178	0.809	0.553
Colombia	1.660	1.766	1.638	1.684	1.178	0.809	0.553
Costa Rica	4.028	1.766	1.638	1.684	1.178	0.809	0.553
Croatia	1.660	1.766	1.638	1.684	1.178	0.809	1.666
Cuba	1.660	0.669	1.638	1.684	1.178	-1.629	-1.289
Denmark	1.660	1.766	1.638	1.684	1.178	0.809	0.553
Dominican Republic	1.660	1.766	1.638	1.684	1.178	0.809	0.553
Ecuador	1.660	1.766	1.638	1.684	1.178	-1.163	-1.585
El Salvador	1.660	1.766	1.638	0.235	0.177	-0.518	-0.536
Finland	1.660	1.766	1.638	1.684	1.178	0.809	0.553
France	1.660	1.766	1.638	1.684	1.178	0.809	0.553
Germany	1.660	1.766	1.638	1.684	1.178	0.809	0.553
Ghana	1.660	1.766	1.638	1.684	1.178	0.809	0.553
Greece	1.660	1.766	1.638	1.684	1.178	0.809	0.553
Guatemala	1.660	1.766	1.638	1.684	1.178	0.809	0.553
Honduras	1.660	1.766	1.638	1.684	0.129	-0.676	0.553
Hungary	1.660	1.766	1.638	1.684	-0.026	-0.472	-0.966
India	1.660	1.766	1.638	1.684	1.178	1.849	0.553
Iraq	1.660	1.766	1.638	1.684	1.178	0.809	0.553
Ireland	1.660	1.766	1.638	1.684	1.178	0.809	0.553
Israel	1.660	1.766	1.638	1.684	1.178	0.809	1.557
Italy	1.660	1.766	1.638	1.684	1.178	0.809	0.553
Japan	1.660	1.766	1.638	1.684	1.178	0.809	0.553
Latvia	1.660	1.766	1.638	1.684	1.178	2.034	2.143
Mexico	1.660	1.766	1.638	1.684	1.178	0.809	0.553
Morocco	1.660	1.766	1.638	1.684	1.178	0.809	0.553
Nepal	1.660	1.766	1.638	1.684	1.178	0.809	0.553
Netherlands	1.660	1.766	1.638	1.684	1.178	0.809	0.553
New Zealand	1.660	1.766	1.638	1.684	1.178	0.809	0.553
Nicaragua	-0.015	0.013	0.055	1.684	0.008	0.809	0.553
Nigeria	1.660	1.766	1.638	1.684	1.178	0.809	0.553
North Macedonia	1.660	1.766	1.638	1.684	1.178	0.809	0.553
Norway	1.660	1.766	1.638	1.684	1.178	-0.346	0.553
Pakistan	1.660	1.766	1.638	1.684	1.178	0.809	0.553
Panama	1.660	1.766	1.638	1.684	1.178	0.809	-1.472
Paraguay	1.660	1.766	1.638	1.684	-0.121	-1.143	-1.850
Peru	1.660	1.766	1.638	1.684	1.178	-0.827	-0.830
Philippines	1.660	1.766	1.638	1.684	1.178	0.809	0.553
Poland	1.660	1.766	1.638	1.684	1.178	0.809	0.553
Romania	1.660	1.766	1.638	1.684	1.178	0.809	0.553
Russia	1.660	1.766	1.638	1.684	1.178	0.809	0.553
Senegal	1.660	1.766	1.638	1.684	1.178	0.809	0.553
Serbia	1.660	1.766	1.638	1.684	1.178	0.809	0.553
Singapore	1.660	1.766	1.638	1.684	1.178	0.809	0.553
Slovakia	1.660	1.766	1.638	1.684	1.178	0.809	0.553
South Africa	1.660	1.766	1.638	1.684	1.178	0.809	0.553
South Korea	1.660	1.766	1.638	1.684	1.178	0.809	0.553
Spain	1.660	1.766	1.638	1.684	1.178	0.809	0.553
Sweden	1.660	1.766	1.638	1.684	1.178	0.809	-0.625
Switzerland	1.660	1.766	1.638	1.684	1.178	0.809	0.553
Taiwan	1.660	1.766	1.638	1.684	1.178	0.809	0.553
Turkey	1.660	1.766	1.638	1.684	1.178	0.809	0.553
Ukraine	0.638	0.462	0.604	0.599	1.178	0.809	1.846
United Kingdom	1.660	1.766	1.638	1.684	1.178	0.809	0.553
United States	1.660	1.766	1.638	1.684	1.178	0.809	0.553
Uruguay	1.660	1.766	1.638	1.684	1.178	-0.511	-0.678
Venezuela	1.660	1.766	1.638	1.684	1.178	-1.094	-1.079

5.2.3. Table S24

Table S24 | Estimated DIF Effects for Factor Loadings

	mac_1	mac_2	mac_3	mac_4	mac_5	mac_6	mac_7
Argentina	0.000	0.000	0.000	0.000	0.000	-1.310	-1.373
Australia	0.000	0.000	0.000	0.000	0.000	0.000	0.000
Austria	0.000	0.000	0.000	0.000	0.000	0.000	0.000
Bangladesh	0.000	0.000	0.000	0.000	-1.678	-1.838	-1.340
Belgium	0.000	0.000	0.000	0.000	0.000	0.000	0.000
Bolivia	0.000	0.000	0.000	0.000	0.000	-1.722	-1.700
Brazil	0.000	0.000	0.000	0.000	0.000	0.000	0.000
Bulgaria	0.000	0.000	0.000	0.000	0.000	0.000	0.000
Canada	0.000	0.000	0.000	0.000	0.000	0.000	0.000
Chile	0.000	0.000	0.000	0.000	-1.373	0.000	0.000
China	0.000	0.000	0.000	0.000	0.000	0.000	0.000
Colombia	0.000	0.000	0.000	0.000	0.000	0.000	0.000
Costa Rica	2.368	0.000	0.000	0.000	0.000	0.000	0.000
Croatia	0.000	0.000	0.000	0.000	0.000	0.000	1.113
Cuba	0.000	-1.097	0.000	0.000	0.000	-2.438	-1.842
Denmark	0.000	0.000	0.000	0.000	0.000	0.000	0.000
Dominican Republic	0.000	0.000	0.000	0.000	0.000	0.000	0.000
Ecuador	0.000	0.000	0.000	0.000	0.000	-1.971	-2.138
El Salvador	0.000	0.000	0.000	-1.448	-1.001	-1.326	-1.089
Finland	0.000	0.000	0.000	0.000	0.000	0.000	0.000
France	0.000	0.000	0.000	0.000	0.000	0.000	0.000
Germany	0.000	0.000	0.000	0.000	0.000	0.000	0.000
Ghana	0.000	0.000	0.000	0.000	0.000	0.000	0.000
Greece	0.000	0.000	0.000	0.000	0.000	0.000	0.000
Guatemala	0.000	0.000	0.000	0.000	0.000	0.000	0.000
Honduras	0.000	0.000	0.000	0.000	-1.049	-1.484	0.000
Hungary	0.000	0.000	0.000	0.000	-1.204	-1.280	-1.519
India	0.000	0.000	0.000	0.000	0.000	1.040	0.000
Iraq	0.000	0.000	0.000	0.000	0.000	0.000	0.000
Ireland	0.000	0.000	0.000	0.000	0.000	0.000	0.000
Israel	0.000	0.000	0.000	0.000	0.000	0.000	1.004
Italy	0.000	0.000	0.000	0.000	0.000	0.000	0.000
Japan	0.000	0.000	0.000	0.000	0.000	0.000	0.000
Latvia	0.000	0.000	0.000	0.000	0.000	1.226	1.590
Mexico	0.000	0.000	0.000	0.000	0.000	0.000	0.000
Morocco	0.000	0.000	0.000	0.000	0.000	0.000	0.000
Nepal	0.000	0.000	0.000	0.000	0.000	0.000	0.000
Netherlands	0.000	0.000	0.000	0.000	0.000	0.000	0.000
New Zealand	0.000	0.000	0.000	0.000	0.000	0.000	0.000
Nicaragua	-1.675	-1.752	-1.583	0.000	-1.170	0.000	0.000
Nigeria	0.000	0.000	0.000	0.000	0.000	0.000	0.000
North Macedonia	0.000	0.000	0.000	0.000	0.000	0.000	0.000
Norway	0.000	0.000	0.000	0.000	0.000	-1.155	0.000
Pakistan	0.000	0.000	0.000	0.000	0.000	0.000	0.000
Panama	0.000	0.000	0.000	0.000	0.000	0.000	-2.025
Paraguay	0.000	0.000	0.000	0.000	-1.298	-1.951	-2.403
Peru	0.000	0.000	0.000	0.000	0.000	-1.636	-1.383
Philippines	0.000	0.000	0.000	0.000	0.000	0.000	0.000
Poland	0.000	0.000	0.000	0.000	0.000	0.000	0.000
Romania	0.000	0.000	0.000	0.000	0.000	0.000	0.000
Russia	0.000	0.000	0.000	0.000	0.000	0.000	0.000
Senegal	0.000	0.000	0.000	0.000	0.000	0.000	0.000
Serbia	0.000	0.000	0.000	0.000	0.000	0.000	0.000
Singapore	0.000	0.000	0.000	0.000	0.000	0.000	0.000
Slovakia	0.000	0.000	0.000	0.000	0.000	0.000	0.000
South Africa	0.000	0.000	0.000	0.000	0.000	0.000	0.000
South Korea	0.000	0.000	0.000	0.000	0.000	0.000	0.000
Spain	0.000	0.000	0.000	0.000	0.000	0.000	0.000
Sweden	0.000	0.000	0.000	0.000	0.000	0.000	-1.178
Switzerland	0.000	0.000	0.000	0.000	0.000	0.000	0.000
Taiwan	0.000	0.000	0.000	0.000	0.000	0.000	0.000
Turkey	0.000	0.000	0.000	0.000	0.000	0.000	0.000
Ukraine	-1.022	-1.303	-1.035	-1.084	0.000	0.000	1.293
United Kingdom	0.000	0.000	0.000	0.000	0.000	0.000	0.000
United States	0.000	0.000	0.000	0.000	0.000	0.000	0.000
Uruguay	0.000	0.000	0.000	0.000	0.000	-1.319	-1.231
Venezuela	0.000	0.000	0.000	0.000	0.000	-1.903	-1.632

5.2.4. Table S25

Table S25 | Summary of results for factor intercepts

Parameter tolerance value	1
Total number of items	469
Number of unique items parameters	73
Percentage of non-invariance parameters	14.1 %
R^2	.98

5.2.5. Table S26

Table S26 | Estimated Item Parameters for Factor Intercepts

	mac_1	mac_2	mac_3	mac_4	mac_5	mac_6	mac_7
Argentina	7.237	8.377	7.764	8.049	4.994	4.230	3.958
Australia	5.898	5.760	6.483	6.161	4.994	5.281	5.259
Austria	5.898	5.760	6.483	6.161	4.994	5.281	5.259
Bangladesh	7.405	5.760	6.483	6.161	4.994	5.281	3.355
Belgium	5.898	5.760	6.483	6.161	4.994	5.281	5.259
Bolivia	5.898	6.861	4.456	6.161	4.994	5.281	5.259
Brazil	5.898	5.760	6.483	6.161	4.994	5.281	5.259
Bulgaria	5.898	5.760	6.483	6.161	4.994	5.281	5.259
Canada	5.898	5.760	6.483	6.161	4.994	5.281	5.259
Chile	5.898	7.080	6.483	6.161	4.994	4.072	3.845
China	5.898	5.760	6.483	6.161	4.994	5.281	5.259
Colombia	5.898	5.760	6.483	6.161	4.994	5.281	5.259
Costa Rica	5.898	8.399	8.283	8.198	3.712	3.426	2.891
Croatia	5.898	5.760	6.483	6.161	4.994	5.281	5.259
Cuba	5.898	7.916	4.825	6.161	4.994	5.281	5.259
Denmark	5.898	5.760	6.483	6.161	4.994	5.281	5.259
Dominican Republic	5.898	5.760	5.066	6.161	4.994	4.156	4.010
Ecuador	5.898	7.245	6.483	6.161	4.994	5.281	5.259
El Salvador	5.898	5.760	6.483	7.913	4.994	5.281	5.259
Finland	5.898	5.760	6.483	6.161	4.994	6.426	7.127
France	5.898	5.760	6.483	6.161	4.994	5.281	5.259
Germany	5.898	5.760	6.483	6.161	4.994	5.281	5.259
Ghana	5.898	5.760	6.483	6.161	6.152	5.281	5.259
Greece	5.898	5.760	6.483	6.161	4.994	5.281	5.259
Guatemala	5.898	5.760	5.286	6.161	4.994	5.281	5.259
Honduras	5.898	5.760	5.028	6.161	4.994	5.281	5.259
Hungary	5.898	5.760	6.483	6.161	4.994	5.281	5.259
India	7.371	5.760	6.483	6.161	4.994	5.281	3.533
Iraq	5.898	5.760	6.483	6.161	4.994	5.281	5.259
Ireland	5.898	5.760	6.483	6.161	4.994	5.281	5.259
Israel	5.898	5.760	6.483	6.161	4.994	5.281	5.259
Italy	5.898	5.760	6.483	6.161	4.994	5.281	5.259
Japan	6.915	5.760	6.483	6.161	4.994	5.281	5.259
Latvia	5.898	5.760	6.483	6.161	4.994	5.281	5.259
Mexico	5.898	5.760	6.483	6.161	4.994	5.281	5.259
Morocco	5.898	5.760	6.483	6.161	4.994	5.281	5.259
Nepal	5.898	5.760	5.287	6.161	4.994	5.281	5.259
Netherlands	5.898	5.760	6.483	6.161	4.994	5.281	5.259
New Zealand	5.898	5.760	6.483	6.161	4.994	5.281	5.259
Nicaragua	8.619	9.393	8.186	-4.588	7.437	2.876	1.602
Nigeria	5.898	5.760	6.483	6.161	4.994	5.281	5.259
North Macedonia	5.898	5.760	6.483	6.161	4.994	5.281	5.259
Norway	5.898	5.760	6.483	6.161	4.994	5.281	5.259
Pakistan	5.898	5.760	6.483	6.161	4.994	5.281	5.259
Panama	5.898	6.975	6.483	6.161	4.994	5.281	7.181
Paraguay	5.898	5.760	6.483	6.161	6.024	4.254	3.696
Peru	5.898	7.019	5.180	6.161	4.994	5.281	5.259
Philippines	5.898	5.760	6.483	6.161	4.994	5.281	4.091
Poland	5.898	5.760	6.483	6.161	4.994	5.281	5.259
Romania	5.898	5.760	6.483	6.161	4.994	5.281	5.259
Russia	5.898	4.488	6.483	6.161	4.994	5.281	5.259
Senegal	5.898	6.784	6.483	6.161	4.994	4.130	3.797
Serbia	4.825	4.337	6.483	6.161	4.994	5.281	5.259
Singapore	5.898	5.760	6.483	6.161	4.994	5.281	5.259
Slovakia	5.898	5.760	6.483	6.161	4.994	5.281	5.259
South Africa	5.898	5.760	6.483	6.161	4.994	5.281	5.259
South Korea	5.898	5.760	6.483	6.161	4.994	5.281	5.259
Spain	5.898	6.942	6.483	6.161	4.994	5.281	5.259
Sweden	5.898	5.760	6.483	6.161	3.790	4.162	5.259
Switzerland	5.898	5.760	6.483	6.161	4.994	5.281	5.259
Taiwan	5.898	5.760	6.483	6.161	4.994	5.281	5.259
Turkey	5.898	5.760	6.483	6.161	4.994	5.281	6.605
Ukraine	5.898	5.760	7.618	6.161	4.994	5.281	5.259
United Kingdom	5.898	5.760	6.483	6.161	4.994	5.281	5.259
United States	5.898	5.760	6.483	6.161	4.994	5.281	5.259
Uruguay	4.834	5.760	6.483	6.161	3.656	4.217	5.259
Venezuela	6.953	5.760	4.766	6.161	4.994	5.281	5.259

5.2.6. Table S27

Table S27 | Estimated DIF Effects for Factor Intercepts

	mac_1	mac_2	mac_3	mac_4	mac_5	mac_6	mac_7
Argentina	1.339	2.617	1.281	1.887	0.000	-1.051	-1.300
Australia	0.000	0.000	0.000	0.000	0.000	0.000	0.000
Austria	0.000	0.000	0.000	0.000	0.000	0.000	0.000
Bangladesh	1.507	0.000	0.000	0.000	0.000	0.000	-1.903
Belgium	0.000	0.000	0.000	0.000	0.000	0.000	0.000
Bolivia	0.000	1.101	-2.027	0.000	0.000	0.000	0.000
Brazil	0.000	0.000	0.000	0.000	0.000	0.000	0.000
Bulgaria	0.000	0.000	0.000	0.000	0.000	0.000	0.000
Canada	0.000	0.000	0.000	0.000	0.000	0.000	0.000
Chile	0.000	1.320	0.000	0.000	0.000	-1.209	-1.413
China	0.000	0.000	0.000	0.000	0.000	0.000	0.000
Colombia	0.000	0.000	0.000	0.000	0.000	0.000	0.000
Costa Rica	0.000	2.640	1.800	2.037	-1.283	-1.855	-2.368
Croatia	0.000	0.000	0.000	0.000	0.000	0.000	0.000
Cuba	0.000	2.156	-1.658	0.000	0.000	0.000	0.000
Denmark	0.000	0.000	0.000	0.000	0.000	0.000	0.000
Dominican Republic	0.000	0.000	-1.417	0.000	0.000	-1.126	-1.249
Ecuador	0.000	1.485	0.000	0.000	0.000	0.000	0.000
El Salvador	0.000	0.000	0.000	1.752	0.000	0.000	0.000
Finland	0.000	0.000	0.000	0.000	0.000	1.144	1.868
France	0.000	0.000	0.000	0.000	0.000	0.000	0.000
Germany	0.000	0.000	0.000	0.000	0.000	0.000	0.000
Ghana	0.000	0.000	0.000	0.000	1.157	0.000	0.000
Greece	0.000	0.000	0.000	0.000	0.000	0.000	0.000
Guatemala	0.000	0.000	-1.198	0.000	0.000	0.000	0.000
Honduras	0.000	0.000	-1.455	0.000	0.000	0.000	0.000
Hungary	0.000	0.000	0.000	0.000	0.000	0.000	0.000
India	1.473	0.000	0.000	0.000	0.000	0.000	-1.726
Iraq	0.000	0.000	0.000	0.000	0.000	0.000	0.000
Ireland	0.000	0.000	0.000	0.000	0.000	0.000	0.000
Israel	0.000	0.000	0.000	0.000	0.000	0.000	0.000
Italy	0.000	0.000	0.000	0.000	0.000	0.000	0.000
Japan	1.017	0.000	0.000	0.000	0.000	0.000	0.000
Latvia	0.000	0.000	0.000	0.000	0.000	0.000	0.000
Mexico	0.000	0.000	0.000	0.000	0.000	0.000	0.000
Morocco	0.000	0.000	0.000	0.000	0.000	0.000	0.000
Nepal	0.000	0.000	-1.196	0.000	0.000	0.000	0.000
Netherlands	0.000	0.000	0.000	0.000	0.000	0.000	0.000
New Zealand	0.000	0.000	0.000	0.000	0.000	0.000	0.000
Nicaragua	2.721	3.633	1.703	-10.749	2.443	-2.405	-3.656
Nigeria	0.000	0.000	0.000	0.000	0.000	0.000	0.000
North Macedonia	0.000	0.000	0.000	0.000	0.000	0.000	0.000
Norway	0.000	0.000	0.000	0.000	0.000	0.000	0.000
Pakistan	0.000	0.000	0.000	0.000	0.000	0.000	0.000
Panama	0.000	1.215	0.000	0.000	0.000	0.000	1.923
Paraguay	0.000	0.000	0.000	0.000	1.029	-1.028	-1.562
Peru	0.000	1.260	-1.304	0.000	0.000	0.000	0.000
Philippines	0.000	0.000	0.000	0.000	0.000	0.000	-1.168
Poland	0.000	0.000	0.000	0.000	0.000	0.000	0.000
Romania	0.000	0.000	0.000	0.000	0.000	0.000	0.000
Russia	0.000	-1.272	0.000	0.000	0.000	0.000	0.000
Senegal	0.000	1.024	0.000	0.000	0.000	-1.151	-1.461
Serbia	-1.073	-1.423	0.000	0.000	0.000	0.000	0.000
Singapore	0.000	0.000	0.000	0.000	0.000	0.000	0.000
Slovakia	0.000	0.000	0.000	0.000	0.000	0.000	0.000
South Africa	0.000	0.000	0.000	0.000	0.000	0.000	0.000
South Korea	0.000	0.000	0.000	0.000	0.000	0.000	0.000
Spain	0.000	1.182	0.000	0.000	0.000	0.000	0.000
Sweden	0.000	0.000	0.000	0.000	-1.204	-1.119	0.000
Switzerland	0.000	0.000	0.000	0.000	0.000	0.000	0.000
Taiwan	0.000	0.000	0.000	0.000	0.000	0.000	0.000
Turkey	0.000	0.000	0.000	0.000	0.000	0.000	1.347
Ukraine	0.000	0.000	1.135	0.000	0.000	0.000	0.000
United Kingdom	0.000	0.000	0.000	0.000	0.000	0.000	0.000
United States	0.000	0.000	0.000	0.000	0.000	0.000	0.000
Uruguay	-1.064	0.000	0.000	0.000	-1.338	-1.064	0.000
Venezuela	1.054	0.000	-1.717	0.000	0.000	0.000	0.000

6. Additional Descriptive Statistics

6.1. Summary statistics of subjective SES measure

6.1.1. Table S28

Table S28 | Summary statistics of subjective SES across all 67 countries

Country	GINI	Mean	SD	Median	Mini	Max
Argentina	41.4	4.67	1.60	4	1	11
Australia	34.4	5.54	2.07	6	1	11
Austria	29.7	4.23	1.65	4	1	11
Bangladesh	32.4	6.28	1.70	6	1	11
Belgium	27.4	4.83	1.58	5	1	10
Bolivia	42.2	4.90	2.06	5	1	9
Brazil	53.9	5.00	1.82	5	1	11
Bulgaria	40.4	4.96	1.49	5	1	10
Canada	33.8	5.90	1.82	6	1	11
Chile	44.4	4.33	1.83	4	1	10
China	38.5	6.64	1.32	6	4	11
Colombia	50.4	4.85	1.56	5	1	11
Costa Rica	48	3.92	1.38	4	2	7
Croatia	30.4	6.00	1.77	6	1	11
Cuba	38	4.60	2.08	4	1	11
Denmark	28.7	5.29	1.77	5	1	11
Dominican Rep.	43.7	5.00	1.91	5	1	11
Ecuador	45.4	4.94	1.68	5	1	10
El Salvador	38.6	4.71	1.44	4.5	2	8
Finland	27.4	5.49	2.04	5	1	11
France	31.6	5.49	1.90	6	1	11
Germany	31.9	5.80	1.71	6	1	11
Ghana	43.5	5.80	1.81	6	1	11
Greece	34.4	5.45	1.47	5	2	11
Guatemala	48.3	4.65	1.64	4.5	1	9
Honduras	52.1	4.83	2.06	4	1	9
Hungary	30.6	6.06	1.66	6	1	11
India	37.8	5.75	2.02	6	1	11
Iraq	29.5	6.13	2.51	6	1	11
Ireland	32.8	5.31	1.66	5	1	11
Israel	39	5.43	1.80	6	1	11
Italy	35.9	5.67	1.89	6	1	11
Japan	32.9	6.35	1.89	6	1	11
Latvia	35.6	5.56	1.83	6	1	11
Mexico	45.4	4.64	1.71	4	1	11

Morocco	39.5	5.10	1.69	5	1	11
Nepal	32.8	5.56	1.74	6	1	11
Netherlands	28.5	4.61	1.72	4	1	11
New Zealand	32.5	5.55	2.01	6	1	11
Nicaragua	46.2	5.63	2.28	5	3	10
Nigeria	43	6.11	1.85	6	1	11
N. Macedonia	34.2	4.86	1.67	5	1	11
Norway	27	5.37	1.69	6	1	11
Pakistan	33.5	4.79	1.84	5	1	11
Panama	49.2	4.28	1.96	4	1	9
Paraguay	46.2	4.94	2.08	6	1	8
Peru	42.8	5.00	1.78	5	1	10
Philippines	44.4	5.59	1.71	6	1	11
Poland	29.7	6.05	1.83	6	1	11
Romania	36	5.88	1.73	6	1	11
Russia	37.5	6.69	1.58	6	1	11
Senegal	40.3	5.63	2.24	6	1	11
Serbia	36.2	5.83	1.71	6	1	11
Singapore	39.8	5.52	1.77	6	1	11
Slovakia	25.2	5.79	1.58	6	1	11
South Africa	63	4.55	1.83	4	1	10
South Korea	31.6	5.61	2.16	6	1	11
Spain	34.7	4.93	1.62	5	1	11
Sweden	28.8	4.85	1.72	5	1	11
Switzerland	32.7	5.40	1.92	6	1	11
Taiwan	33.8	5.98	1.74	6	1	11
Turkey	41.9	5.64	2.03	6	1	11
Ukraine	26.1	6.10	1.63	6	1	11
UK	34.8	5.62	1.75	6	2	11
US	41.4	5.28	2.33	6	1	11
Uruguay	39.7	4.41	1.27	4	2	7
Venezuela	46.9	5.77	2.09	6	2	11

6.1.2. Table S29

Table S29 | Summary statistics of subjective SES status across nationally representative sample

Country	GINI	Mean	SD	Median	Min	Max
Australia	34.4	5.54	2.07	6	1	11
Austria	29.7	4.23	1.65	4	1	11
Canada	33.8	5.90	1.82	6	1	11
China	38.5	6.64	1.32	6	4	11
Croatia	30.4	6.00	1.77	6	1	11

Denmark	28.7	5.29	1.77	5	1	11
France	31.6	5.49	1.90	6	1	11
Germany	31.9	5.80	1.71	6	1	11
Hungary	30.6	6.06	1.66	6	1	11
Israel	39	5.43	1.80	6	1	11
Japan	32.9	6.35	1.89	6	1	11
Latvia	35.6	5.56	1.83	6	1	11
Netherlands	28.5	4.61	1.72	4	1	11
New Zealand	32.5	5.55	2.01	6	1	11
Nigeria	43	6.11	1.85	6	1	11
Norway	27	5.37	1.69	6	1	11
Philippines	44.4	5.59	1.71	6	1	11
Poland	29.7	6.05	1.83	6	1	11
Singapore	39.8	5.52	1.77	6	1	11
Slovakia	25.2	5.79	1.58	6	1	11
South Korea	31.6	5.61	2.16	6	1	11
Spain	34.7	4.93	1.62	5	1	11
Sweden	28.8	4.85	1.72	5	1	11
Switzerland	32.7	5.40	1.92	6	1	11
Taiwan	33.8	5.98	1.74	6	1	11
Turkey	41.9	5.64	2.03	6	1	11
UK	34.8	5.62	1.75	6	2	11
US	41.4	5.28	2.33	6	1	11

END OF SUPPLEMENTARY INFORMATION
