# Supplementary material for: Subjective socioeconomic status and income inequality are associated with self-reported morality across 67 countries
Source: Nat Commun. 2023 Sep 6;14:5453. doi: 10.1038/s41467-023-41007-0 (PMC10482940; doi:10.1038/s41467-023-41007-0)
Supplement: Supplementary file 3 — Description of Additional Supplementary Files [file 41467_2023_41007_MOESM3_ESM.pdf]

Title: Supplementary Data 1

Description: Country-level correlations between subjective socioeconomic status (SES) and the four indicators of self-reported morality (Moral identity, Morality-as-Cooperation, Moral Circle and Prosocial Intention) across the 67 countries.
