# Supplementary material for: Subjective socioeconomic status and income inequality are associated with self-reported morality across 67 countries
Source: Nat Commun. 2023 Sep 6;14:5453. doi: 10.1038/s41467-023-41007-0 (PMC10482940; doi:10.1038/s41467-023-41007-0)
Supplement: Supplementary file 4 — Reporting Summary [file 41467_2023_41007_MOESM4_ESM.pdf]

nature portfolio

Corresponding author(s): Christian T. Elbaek

Last updated by author(s): Jul 31, 2023

Reporting Summary

Nature Portfolio wishes to improve the reproducibility of the work that we publish. This form provides structure for consistency and transparency in reporting. For further information on Nature Portfolio policies, see our Editorial Policies and the Editorial Policy Checklist.

Statistics

For all statistical analyses, confirm that the following items are present in the figure legend, table legend, main text, or Methods section.

n/a	Confirmed	
☐	☒	The exact sample size (*n*) for each experimental group/condition, given as a discrete number and unit of measurement
☐	☒	A statement on whether measurements were taken from distinct samples or whether the same sample was measured repeatedly
☐	☒	The statistical test(s) used AND whether they are one- or two-sided<br>*Only common tests should be described solely by name; describe more complex techniques in the Methods section.*
☐	☒	A description of all covariates tested
☐	☒	A description of any assumptions or corrections, such as tests of normality and adjustment for multiple comparisons
☐	☒	A full description of the statistical parameters including central tendency (e.g. means) or other basic estimates (e.g. regression coefficient) AND variation (e.g. standard deviation) or associated estimates of uncertainty (e.g. confidence intervals)
☐	☒	For null hypothesis testing, the test statistic (e.g. *F*, *t*, *r*) with confidence intervals, effect sizes, degrees of freedom and *P* value noted<br>*Give P values as exact values whenever suitable.*
☒	☐	For Bayesian analysis, information on the choice of priors and Markov chain Monte Carlo settings
☐	☒	For hierarchical and complex designs, identification of the appropriate level for tests and full reporting of outcomes
☐	☒	Estimates of effect sizes (e.g. Cohen's *d*, Pearson's *r*), indicating how they were calculated

Our web collection on statistics for biologists contains articles on many of the points above.

Software and code

Policy information about availability of computer code

Data collection	The data were obtained from the International Collaboration on Social & Moral Psychology of COVID-19 (ICSMP): https://icsmp-covid19.netlify.app/index.html. The original survey of the ICSMP project was coded in the survey software Qualtrics. The custom code developed for this project can be accessed on OSF: https://osf.io/dxvmk/
Data analysis	We used the open-source statistical environment R (version 4.0.3) to conduct all statistical analysis.

For manuscripts utilizing custom algorithms or software that are central to the research but not yet described in published literature, software must be made available to editors and reviewers. We strongly encourage code deposition in a community repository (e.g. GitHub). See the Nature Portfolio guidelines for submitting code & software for further information.

Data

Policy information about availability of data

All manuscripts must include a data availability statement. This statement should provide the following information, where applicable:

- Accession codes, unique identifiers, or web links for publicly available datasets
- A description of any restrictions on data availability
- For clinical datasets or third party data, please ensure that the statement adheres to our policy

The ICSMP data can accessed on the OSF page of the original article: https://osf.io/y7ckt/. The dataset containing the GINI Indexes and net-adjusted household income for every country in the study is available on the OSF page of this study: https://osf.io/dxvmk/. Data on the GINI coefficients for all countries except Taiwan, Cuba, New Zealand and Singapore were obtained from the World Bank and be accessed at: https://data.worldbank.org/indicator/SI.POV.GINI. The GINI coefficient

for Taiwan was obtained from Statista and can be accessed at: https://www.statista.com/statistics/922574/taiwan-gini-index/. The GINI coefficient for Cuba was obtained from Reuters and can be accessed at: https://www.reuters.com/article/us-cuba-reform-inequality/cuba-grapples-with-grow-ing-inequality-idUSN1033501920080410. The GINI coefficient for New Zealand was obtained from Knoema and be accessed at: https://knoema.com/atlas/New-Zealand/topics/Poverty/Income-Inequality/GINI-index. The GINI index for Singapore was obtained from Knoema and can be accessed at: https://knoema.com/atlas/Singapore/GINI-index. Region names for the region classification were obtained through the R package countrycode and be accessed by running the package described in the following link: https://cran.r-project.org/web/packages/countrycode/countrycode.pdf. Data on Adjusted Disposable Net-Income were obtained from the World Bank and be accessed at: https://data.worldbank.org/indicator/NY.ADJ.NNTY.PC.CD.

Research involving human participants, their data, or biological material

Policy information about studies with human participants or human data. See also policy information about sex, gender (identity/presentation), and sexual orientation and race, ethnicity and racism.

Reporting on sex and gender	We used the term gender in the current research. Gender was not considered in the study design, but was used as covariate in all reported models in the manuscript. Gender is self-reported. A total of 167 individuals reported their gender identification as "Other." We excluded these participants from our formal analysis to reduce the risk that this category of the covariate would inflate the results obtained from our models, given that the "Other" category was too infrequently represented in the data for meaningful country comparisons. The gender distribution of our final sample was 52% female and 48% male.
Reporting on race, ethnicity, or other socially relevant groupings	We use the term "Subjective socioeconomic status (SES)" to classify people's subjective socioeconomic status. We form this variable based on participants self-report on the MacArthur ladder scale. For the MacArthur scale, individuals are asked to place themselves on a ladder with 11 steps, where selecting the lowest step (1) indicates that you would place yourself among the people with the least financial resources, least education, and least attractive jobs in your respective country, while placing yourself on the top of the ladder (11) indicates that you place yourself among the people with the most financial resources, best education, and most attractive jobs. In our analysis we control for age and gender and all models are cross-validated for robustness.
Population characteristics	The overall sample contains 50,396 individuals from 67 countries. Of the 67 countries, 28 countries used fully representative samples with respect to gender and age, and 44 countries included more than 500 participants. Mean age was 43 years and 52% of participants were female.
Recruitment	Participants were recruited through panel agencies and online platforms. We do not consider any potential self-selection bias to be present, as also argued in the original publication building on the same data (see Van Bavel, J. J., Cichocka, A., Capraro, V., Sjåstad, H., Nezlek, J. B., Pavlović, T., Alfano, M., Gelfand, M. J., Azevedo, F., Birtel, M. D., Cislak, A., Lockwood, P. L., Ross, R. M., Abts, K., Agadullina, E., Aruta, J. J. B., Besharati, S. N., Bor, A., Choma, B. L., . . . Boggio, P. S. (2022). National identity predicts public health support during a global pandemic. Nature Communications, 13(1), 517. https://doi.org/10.1038/s41467-021-27668-9).
Ethics oversight	The project received ethical approval from the institutional review board at the University of Kent (ID 202015872211976468) and informed consent was obtained from all participants prior to their voluntary participation in the study.

Note that full information on the approval of the study protocol must also be provided in the manuscript.

Field-specific reporting

Please select the one below that is the best fit for your research. If you are not sure, read the appropriate sections before making your selection.

☐ Life sciences ☒ Behavioural & social sciences ☐ Ecological, evolutionary & environmental sciences

For a reference copy of the document with all sections, see nature.com/documents/nr-reporting-summary-flat.pdf

Behavioural & social sciences study design

All studies must disclose on these points even when the disclosure is negative.

Study description	The study is a quantitative study based on empirical, self-reported, partly representative data from 67 nations across the world. Using advanced quantitative methods, the study investigates the association between individual- and macro-levels of economic inequality and human morality.
Research sample	The research sample consists of self-reported demographics and social and moral psychology data from 50,396 individuals from 67 countries and 5 different regions of the world. Of all included countries, 28 are fully representative samples with respect to gender and age, whereas 44 consist of more than 500 participants. Participants' mean age was 43 years and 52% of participants were females. We chose this sample because we were a part of collecting the original dataset and thus had prior access to these data.
Sampling strategy	The sample which makes up the dataset was recruited by over 200 researchers from around the world. No statistical sample size calculation was performed but every participating research team was asked to collect a sample of at least 500 participants. Convenience sampling was used for non-representative samples (N = 39 countries). Stratified sampling was used for nationally representative samples (N = 28 countries). Data were collected through professional panel agencies or online platforms (e.g., Prolific Academic).

Data collection	The data were collected between April and May 2020 and were administered using an online survey. Each national team responsible for collecting data in their country translated the English survey into their country's national language using the standard forward-backward translation method. The instrument used to record the data can accessed at the OSF page of the original study: https://osf.io/y7ckt/. We are unable to know whether participants were alone (or with family or friends) when taking part in the survey. To the best of our knowledge, no scholar from the research team was physically present when participants took part in the survey.
Timing	The data were collected between April 22, 2020, and May 30, 2020.
Data exclusions	A total of 53,269 participants answered the survey. Of these, 2,049 were excluded for not having completed the full survey, and 131 were excluded for being younger than 18 y/o or older than 100 y/o. Furthermore, we removed 526 participants who failed attention checks and 167 participants who reported "Other" as their gender identification. For gender identification, we excluded these participants from our formal analysis to reduce the risk that this category of the covariate would inflate the results obtained from our models, given that the "Other" category was too infrequently represented in the data for meaningful country comparisons. This resulted in a final sample of 50,396 participants.
Non-participation	2,049 participants were excluded for not having completed the full survey.
Randomization	Scale order was randomized for every participant.

Reporting for specific materials, systems and methods

We require information from authors about some types of materials, experimental systems and methods used in many studies. Here, indicate whether each material, system or method listed is relevant to your study. If you are not sure if a list item applies to your research, read the appropriate section before selecting a response.

Materials & experimental systems

n/a	Involved in the study
☒	☐ Antibodies
☒	☐ Eukaryotic cell lines
☒	☐ Palaeontology and archaeology
☒	☐ Animals and other organisms
☒	☐ Clinical data
☒	☐ Dual use research of concern
☒	☐ Plants

Methods

n/a	Involved in the study
☒	☐ ChIP-seq
☒	☐ Flow cytometry
☒	☐ MRI-based neuroimaging
